# The Transcriptomic Landscape of Prostate Cancer Development and Progression: An Integrative Analysis

**DOI:** 10.3390/cancers13020345

**Published:** 2021-01-19

**Authors:** Jacek Marzec, Helen Ross-Adams, Stefano Pirrò, Jun Wang, Yanan Zhu, Xueying Mao, Emanuela Gadaleta, Amar S. Ahmad, Bernard V. North, Solène-Florence Kammerer-Jacquet, Elzbieta Stankiewicz, Sakunthala C. Kudahetti, Luis Beltran, Guoping Ren, Daniel M. Berney, Yong-Jie Lu, Claude Chelala

**Affiliations:** 1 Bioinformatics Unit, Centre for Cancer Biomarkers and Biotherapeutics, Barts Cancer Institute, Queen Mary University of London, London EC1M 6BQ, UK; jacek.marzec@unimelb.edu.au (J.M.); s.pirro@ucl.ac.uk (S.P.); j.a.wang@qmul.ac.uk (J.W.); e.gadaleta@qmul.ac.uk (E.G.); 2 Centre for Cancer Biomarkers and Biotherapeutics, Barts Cancer Institute, Queen Mary University of London, London EC1M 6BQ, UK; yanan.zhu@qmul.ac.uk (Y.Z.); x.mao@qmul.ac.uk (X.M.); soleneflorence.kammerer-jacquet@chu-rennes.fr (S.-F.K.-J.); e.stankiewicz@qmul.ac.uk (E.S.); s.kudahetti@qmul.ac.uk (S.C.K.); d.m.berney@qmul.ac.uk (D.M.B.); y.j.lu@qmul.ac.uk (Y.-J.L.); 3 Centre for Cancer Prevention, Wolfson Institute of Preventive Medicine, Barts and the London School of Medicine, Queen Mary University of London, London EC1M 6BQ, UK; amar.ahmad@qmul.ac.uk (A.S.A.); b.v.north@qmul.ac.uk (B.V.N.); 4 Department of Pathology, Barts Health NHS, London E1 F1R, UK; luis.beltran@bartsandthelondon.nhs.uk; 5 Department of Pathology, The First Affiliated Hospital, Zhejiang University Medical College, Hangzhou 310058, China; 1190024@zju.edu.cn; 6 Centre for Computational Biology, Life Sciences Initiative, Queen Mary University London, London EC1M 6BQ, UK

**Keywords:** prostate cancer, tumorigenesis, mRNA, RNAseq, transcriptomic, data integration

## Abstract

**Simple Summary:**

There is a tremendous amount of gene expression information available for prostate cancer, but very few tools exist to combine the disparate datasets generated across sample types and technical platforms. We present a method of integrating different types of expression data from different study cohorts to increase analytic power, and improve our understanding of the molecular changes underlying the development and progression of prostate cancer from normal to advanced disease. Using this approach, we identified nine additional disease stage-specific candidate genes with prognostic significance, which were not identified in any one study alone. We have developed a free online tool summarizing our results, and making the complete combined dataset available for further translational research.

**Abstract:**

Next-generation sequencing of primary tumors is now standard for transcriptomic studies, but microarray-based data still constitute the majority of available information on other clinically valuable samples, including archive material. Using prostate cancer (PC) as a model, we developed a robust analytical framework to integrate data across different technical platforms and disease subtypes to connect distinct disease stages and reveal potentially relevant genes not identifiable from single studies alone. We reconstructed the molecular profile of PC to yield the first comprehensive insight into its development, by tracking changes in mRNA levels from normal prostate to high-grade prostatic intraepithelial neoplasia, and metastatic disease. A total of nine previously unreported stage-specific candidate genes with prognostic significance were also found. Here, we integrate gene expression data from disparate sample types, disease stages and technical platforms into one coherent whole, to give a global view of the expression changes associated with the development and progression of PC from normal tissue through to metastatic disease. Summary and individual data are available online at the Prostate Integrative Expression Database (PIXdb), a user-friendly interface designed for clinicians and laboratory researchers to facilitate translational research.

## 1. Introduction

Prostate cancer (PC) is the second most common cancer diagnosis in men worldwide, and the fifth leading cause of cancer-related death [1]. It is clinically and pathologically heterogeneous, presenting a challenge to identifying robust molecular biomarkers for patient management. Although many independent studies have investigated the molecular mechanisms of PC (reviewed in [2]), none so far has presented a global view of the transcriptomics landscape linking key molecular events across different PC stages. The limited statistical power of individual studies, different experimental and analytical parameters, differences in tumor tissue content, and inter- and intra-tumor heterogeneity contribute to the poor overlap between currently available molecular signatures [3]. Furthermore, a significant challenge to effective translational research more generally, is that the sheer volume of ‘-omics’ data available from individual studies is not matched by appropriate tools to easily synthesize disparate datasets, from different disease stages and generated on different platforms, into a coherent whole.

PC has a long natural history and can initiate from disrupted prostate epithelium to develop over many decades. While high-grade prostatic intra-epithelial neoplasia (HGPIN) is recognized as a precursor of cancer, it is morphologically distinct and may remain stable for a long time or never progress [4]; its molecular relationship with primary tumor remains unclear. This makes cross-study analysis an attractive approach to reconcile distinct gene expression signatures from separate studies and to identify genetic alterations not evident from individual experiments, leading to more comprehensive biological insights.

While next-generation sequencing (NGS) technologies are now the technique of choice in transcriptomic studies, reflected in the substantial increase in RNA sequencing (RNAseq) datasets in public repositories, such studies have generally focused on primary tumors following radical prostatectomy (e.g., The Cancer Genome Atlas (TCGA) and International Cancer Genome Consortium (ICGC)). On the other hand, microarray-based data still constitute the majority of publicly-available datasets and is often the only information available about other, equally important, clinically valuable samples (e.g., benign, HGPIN or metastatic tissues) or experimental types (e.g., treatment effects). Therefore, the integration of data from various NGS and microarray platforms and archived or historic sample collections presents an excellent opportunity to maximize research output. By tracking changes in mRNA abundance levels from normal prostate through to HGPIN, localized tumors and finally metastatic disease, in combination with molecular pathway and survival analyses, we define the transcriptional landscape of PC to help elucidate the molecular mechanisms driving the development and progression of this disease.

Compared to meta-analysis, integrating data from separate studies at the raw mRNA abundance level can significantly increase the number of genuine gene expression changes identified. Raw data integration also allows for quality checks of preliminary data, a vital step in integrative analysis, as quality-related problems amplify during downstream data exploration. Furthermore, the increase in sample size by direct data combination increases the statistical power to obtain more precise estimates of gene expression differentials and facilitates the assessment of the overall heterogeneity estimate, resulting in lower false discovery rates. Several tools for data integration have been developed for cross-study analyses. However, each has limitations that preclude the exploration of available transcriptomics data to their full potential. Some resources offer integration of expression data derived solely from the same platform, which narrows the choice of deposited data appropriate for the analysis; other methods use transformation or normalization algorithms to facilitate processing of data from different platforms, but only for those genes present across all technologies. Indeed, inclusion of data from different platforms presenting diverse genomic coverage often reduces the overlap of features eligible for downstream analysis, resulting in the loss of information about potentially important genes. 

Here we present a large multi-cohort analysis of PC mRNA abundance profiles, including 1488 profiles from two RNAseq and 18 array-based datasets from 8 different platforms. Using these, we developed a cross-platform data integration framework to establish a molecular alteration map (MAM) of PC development and progression. The identification of established PC risk genes and pathways demonstrates the reliability of our approach. The collected and integrated data, along with analytical and visualization tools to explore the largest set of high-quality PC-related expression profiles, are freely available online at the Prostate Integrative Expression Database (PIXdb) (www.pixdb.org.uk).

## 2. Materials and Methods

### 2.1. Source Data

**Figure 1:**
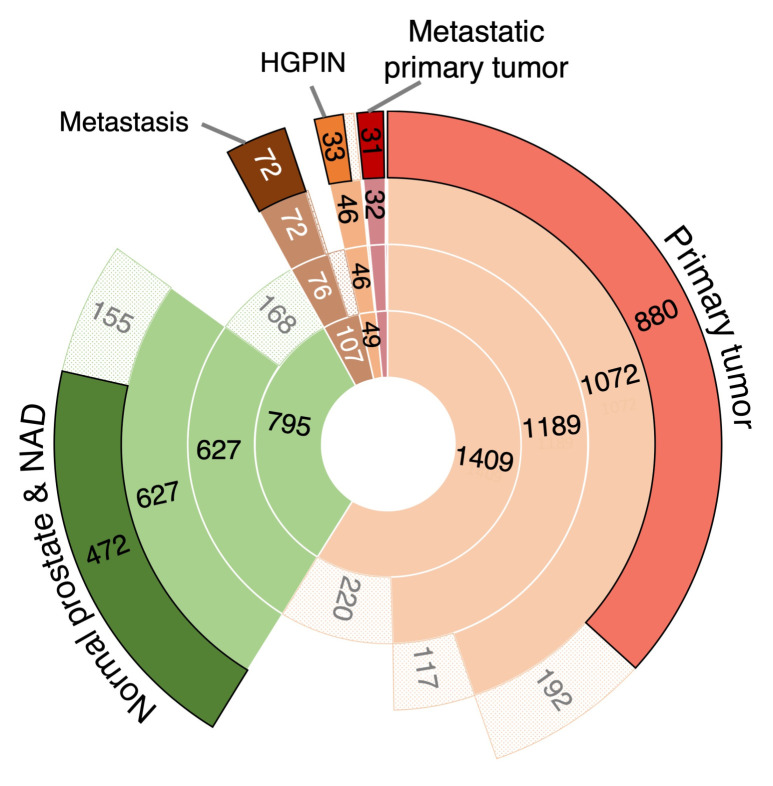
Transcriptomic profiles from a range of prostate tissue types were used in an integrative analysis. The chart presents collated prostate expression profiles and results of the individual quality control steps. The most inner circle corresponds to the number of collected samples representing various tissue types. The second inner, middle, and outer circles reflect the number of samples remaining after filtering based on initial quality control, estimated tumor tissue content, as well as principal component analysis and clustering, respectively (see also Methods and Table S2). The pale areas indicate the proportion of samples filtered out in each biological group in individual filtering steps. 1488 transcriptomic profiles from histologically non-malignant tissues (normal and normal adjacent to tumor; NAD, HGPIN, primary tumor without metastases (primary tumor), primary tumor from patients with metastatic disease (metastatic primary tumor), and metastatic tissues (metastasis), were analyzed.

**Figure 2:**
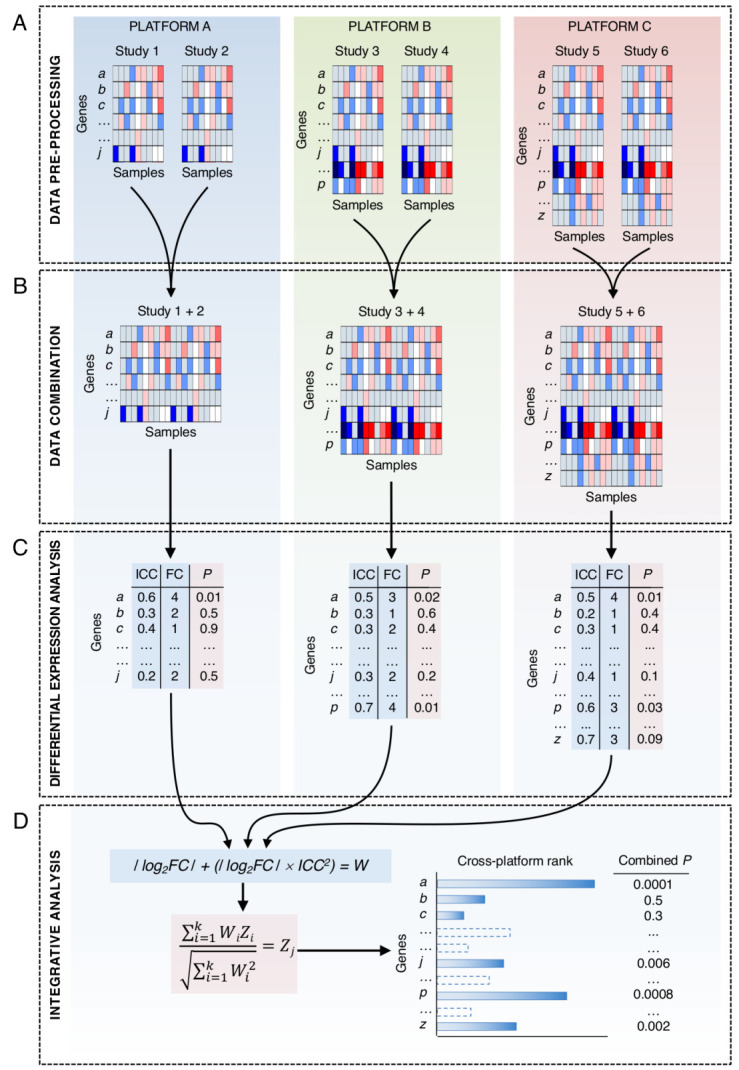
The cross-platform integrative analysis workflow. (**A**) Pre-processing of multi-study expression data generated using different technologies and covering various sets of genes. (**B**) Combination step including normalization and batch-effect adjustment of expression data derived from the same platform and containing the same set of genes. (**C**) Per-platform differential expression analysis outputting fold-change and p-value (FC, P), and integrative correlation coefficient (ICC) for each gene. (**D**) Application of the integrative analysis model, based on the weighted Stouffer’s algorithm, to compute combined *p*-values and determine the cross-platform gene rankings (detailed in Methods).

PC mRNA expression datasets generated on any commercially available microarray platform or by RNASeq were identified from the literature, Gene Expression Omnibus (GEO), Sequence Read Archive (SRA), and ArrayExpress data repositories. No custom arrays were included, and only studies involving untreated biological types were considered, i.e., tumor, normal, benign, HGPIN, metastatic. Datasets publicly available at December 2013 were considered for inclusion in this study. No studies using FFPE samples were used. The 3 RNAseq and 21 array-based PC-related gene expression datasets, generated across 11 different platforms, were identified for inclusion in this study (Supplementary Table S1). In total, 2396 transcriptomics profiles from 795 histologically non-malignant (130 healthy or benign prostatic hyperplasia (BPH) and 665 normal adjacent to tumor (NAD) prostate tissues, 49 HGPIN samples, 1409 samples from patients with primary PC without metastasis (primary tumor), 36 samples from primary PC biopsies from patients with metastasis (metastatic primary tumor) and 107 samples from metastatic PC tissues (metastasis) were collated (Figure 1, Table S1). Clinical information on biochemical recurrence (BCR)-free survival, prostate-specific antigen (PSA) level, Gleason score and tumor stage was also extracted where available. Since the datasets were curated from public repositories providing minimal or no systematic quality assessment, we set stringent quality criteria to ensure the robustness of input data for subsequent analysis. Only those passing all quality controls Table S2) were used in the analytical framework for cross-platform integrative analysis presented in Figure 2 and described below. A total of two datasets were discarded entirely at the initial quality control (QC) stage due to poor overall quality. Subsequent principal component analysis (PCA) and cluster-based data inspection further eliminated two whole datasets (*n* = 258) due to unexplained data variation. The data processing steps, including QC, pre-processing, summarization and integrative analysis, are presented in Supplementary Figure S1. 

### 2.2. Data QC

The quality of array-based datasets was assessed separately using aroma.affymetrix R package for Affymetrix exon arrays and arrayQualityMetrics, and simpleaffy Bioconductor packages for 3′IVT Affymetrix and Illumina microarrays. Aberrant arrays were identified by means of arrayMvout multivariate outlier detection tool implemented in Bioconductor project. The raw RNAseq FASTQ files were examined using FastQC quality control tool.

### 2.3. Non-Malignant Tissue Assessment

To evaluate potential differences at molecular level between healthy prostate, BPH or NAD tissues, PCA was performed using dataset GSE17951, and three sets of data from GSE6919, which together contain expression profiles from each tissue type (Supplementary Figure S2A). Unsupervised hierarchical clustering identified four NADs clustering with tumor (Supplementary Figure S2B), which were removed. This analysis also revealed clear differences in normal and NAD expression profiles compared to primary tumor and metastatic samples, so normal, BPH and remaining NAD samples were subsequently combined into one control group for downstream analyses.

### 2.4. Tumor Content Estimation

Most curated data was acquired from surgically resected bulk tissues that contain an admixture of cell types, and for which tumor content was not typically reported. To reduce the variability effect from tissue types in bulk samples, the collected array expression data were assessed using CellPred algorithm. A 250-gene model was used for in silico prediction of tissue components in samples processed using Affymetrix Human Genome U133 Plus 2.0, U133A and U95Av2 array platforms (12 datasets). Based on the overall distributions of tumor tissue percentage estimated across analyzed datasets, a threshold of 40% was set as minimum acceptable tumor tissue content (Supplementary Figure S2C). Tumor content of included RNAseq datasets was taken from reported histopathology estimates; the same 40% threshold was applied.

### 2.5. Stromal Genes Filtering

A list of consistently deregulated stroma-specific genes was compiled from three resources: (1) 869-gene stroma signature [5], (2) 139 stromal genes used by ESTIMATE, (3) 179 stromal genes in GSE20758 (Figure S2D). All 1 032 suggested stroma-associated genes were filtered out prior to data integration, for a more reliable prostate-specific signature. 

### 2.6. Biological and Laboratory Effects

Cross-study data derived from the same platform were combined and adjusted for batch effects with ComBat. PCA identified key components of variability in the combined and batch effects-adjusted mRNA expression data, and showed that the observed variance correlated with the biological factors (Figure S2E). 

The extent of biological and laboratory effects was also assessed by inspecting average similarities between samples from different studies but the same biological group (e.g., tumor), and samples from the same study but different biological groups (e.g., tumor or benign). In each case, biological effects were significantly stronger than dataset effects (Kolmogorov–Smirnov *p* values < 1 × 10^−10^; Supplementary Figure S2F).

### 2.7. Microarray and RNAseq Data Pre-Processing

Microarray and RNAseq data were pre-processed to unify array probes and sequencing read annotations to increase cross-platform concordance. Microarray probe alignments to gene sequences were retrieved from Ensembl database and used to increase cross-platform concordance by removing probes with insufficient specificity. Probe re-mapping and filtering according to most recent genome sequence annotation was performed to improve the interpretation of biological results derived from microarrays. Gene expression data obtained from the same platform were normalized collectively to minimize batch effects. Data from Affymetrix and Illumina arrays were normalized across samples using GC-Robust Multi-array Average (RMA) and Robust Spline Normalization (RSN), respectively. To unify microarray probe and sequencing read annotations across platforms, multiple probes mapping to the same gene were compiled to build ‘one-probe-to-one-gene’ relational model by selecting probes (Illumina) or probesets (Affymetrix) with the highest standard deviation across samples. 

Raw sequencing data were aligned to human genome (GRCh38) with Bowtie 2 (version 2.1.0) and further summarized with HTSeq Gene read-count data were subjected to conditional quantile normalization (CQN) to remove systematic bias introduced by non-linear guanine-cytosine content and gene length effects. A voom method was applied to estimate the mean-variance relationship and transform the sequencing read counts to log-scale with associated precision weights normalized for sequence depth.

The Ensembl release 90 was used to unify microarray probes and sequencing reads annotations across datasets. RNAseq, Affymetrix Human Exon 1.0 ST, Affymetrix Human Genome U133 Plus 2.0 and Illumina HumanHT-12 V3.0 Expression Beadchip arrays (comprehensive platforms), include most genes present in all other platforms.

### 2.8. Data Integration and Molecular Alterations Map Assembly

Combined and batch effects-corrected data from the same platform were subjected to differential expression analysis using limma. For each platform and biological group comparison (primary tumor vs. normal prostate, HGPIN vs. normal prostate, primary tumor vs. HGPIN, metastatic primary tumor vs. primary tumor, metastasized vs. primary tumor), the expression fold-changes (FC) and *p*-values were computed for all genes. False discovery rate *p*-values were corrected for multiple testing by Benjamini–Hochberg.

Cross-platform data integration (Figure 2) was performed in two steps for each group comparison. First, cross-platform reproducibility for each gene was estimated by calculating the integrative correlation coefficient (ICC) with MergeMaid. Then, *p*-values were combined across platforms using Stouffer’s method with FC values and ICCs used as weighting factors to account for the change in magnitude and expression measurement reproducibility, and to ensure the top rankings captured consistent expression patterns across samples and platforms (Supplementary Figure S3A). Weighting score W for gene a in platform A was estimated as

W_A_ = |log_2_FC_a_| + (|log_2_FC_a_| × ICC_a_^2^)

where log_2_FC_a_ and ICC_a_ indicate the log_2_ FC value and the ICC for a given gene a, respectively, as measured in platform A.

Integration-driven discovery rates (IDR) were computed, to evaluate the impact of integrative analysis on the results. Briefly, IDR estimates the fraction of significant genes detected in the combined results that are not detected in any of the individual platforms for a given significance threshold in Z-score scale (Supplementary Figure S3B). Each Z-score was calculated as the ratio of average effect size over its standard error and represents the statistical significance of the differential expression across multiple experiments.

To determine the number of genes to be used as hallmark of each disease stage, cross-datasets concordance was computed for individual biological comparisons by examining the fraction of genes with log_2_FC > 1 or < −1 in at least half of the datasets (consistently deregulated genes) for the cumulative number of top-k ranked genes. No comparisons involving HGPINs were considered, since only one dataset contained samples with this phenotype. Subsequently, the cross-dataset concordances were plotted against gene rank to determine a threshold close to the points where the plotted lines cross, to ensure that a similar fraction of consistently deregulated genes was in the top-k ranked gene set for each comparison (Supplementary Figure S3C). The threshold was set at 500, representing approximately 60% of consistently deregulated genes in each comparison across datasets. The overlaps between top 500 ranked gene sets were determined to evaluate their logic relationships, and expression changes associated with respective PC stages were tracked to construct a molecular alteration map (MAM). 

### 2.9. Canonical Pathways Analysis

Significant expression changes observed in respective sections of assembled MAM were mapped to canonical pathways using Ingenuity Pathway Analysis (IPA) (Qiagen). For further evidence of pathway enrichment, IPA results were complemented with three additional functional annotation resources: Kyoto Encyclopedia of Genes and Genomes (KEGG), Protein Analysis Through Evolutionary Relationships (PANTHER) and Reactome using Database for Annotation, Visualization and Integrated Discovery (DAVID). Data mining was performed using the same statistical test and criteria for identification of significantly enriched pathways (Fisher’s exact test; *p*-value < 0.05).

### 2.10. Survival Analysis

A univariate Cox proportional hazards regression was applied to available TCGA survival data that passed QC. 387 TCGA samples were assigned to low and high-risk groups based on mRNA expression intensities of the selected gene, using the best-performing cut-off algorithm. In brief, for successive percentiles of expression between the lower and upper quartiles the log-rank test *p*-values were computed and the best performing threshold was used as the final cut-off in regression analysis. Relationships are presented as Kaplan-Meier plots. Hazard ratios, 95% confidence intervals and associated log rank *p*-values were determined. 

### 2.11. Experimental Validation—Patient Samples 

A total of 29 patient samples with 87 matched PC, HGPIN, and benign tissues were identified from formalin-fixed paraffin embedded (FFPE) sections from trans-urethral resection of the prostate (TURP) material from Barts Cancer Institute Orchid Tissue Bank, UK, under license number 12,199. Similarly, matched tumor and benign tissues were macro-dissected from FFPE sections from 25 patients with localized disease. A total of 18 patients with matched PC and benign FFPE tissues from TURP, and 3 patients with matched tumor, normal and HGPIN FFPE tissues were selected for study at the Department of Pathology, The First Affiliated Hospital, Zhejiang University Medical College, Hangzhou, China. Samples were assessed for inclusion by histopathologists S.-F.K.-J., D.M.B., L.B. and G.R. All patients provided informed consent (fresh tissues; FFPE samples used were excess to diagnostic requirements). Studies were approved by the East London and City Research Ethics committee (ref 09/H0704/4+5) and the ethical committee of the First Affiliated Hospital, Zhejiang University Medical College, China, approval number 2102–2142. Gene expression targets were tested in either the N/T or N/HGPIN/T cohorts, depending on their associated MAM section from the integrative analysis.

### 2.12. Experimental Validation—quantitative PCR(qPCR) Analysis

mRNA was extracted from two 10 µm FFPE tissue sections per patient, using a modified Qiazol method and mRNEasy mini kits (Qiagen). Briefly, slides were de-waxed in xylene, before either tumor, benign or HGPIN tissues were macro-dissected and re-suspended in 100 µL extraction buffer (100 mM NaCl, 1 mM EDTA pH 8.0, 10 mM Tris-HCL pH 8.0, 0.5% SDS). Samples were incubated with 20 mg/mL Proteinase K at 50 °C overnight, then mechanically lysed in Qiazol reagent at room temperature, combined with chloroform and separated by high-speed centrifugation for 5 min. The supernatant was combined with 100% ethanol to precipitate mRNA, and isolated by centrifugation through a spin column (Qiagen). Trapped mRNAs were washed with 70% ethanol and salt solutions, according to manufacturer’s instructions, and finally eluted in nuclease-free water. Sample concentration and purity was measured by Qubit (ThermoFisher, Waltham, MA, USA) and Agilent Bioanalyzer 2100. 

RNA was reverse transcribed (SuperScript II, ThermoFisher, Waltham, MA, USA), and assayed in triplicate at each gene target using the 96 × 96 Fluidigm Biomark integrated fluidic cartridge platform and commercially validated TaqMan assays (Supplementary Table S3) according to manufacturer’s instructions. Each reaction plate included four reference cDNA samples, derived from a local, anonymized benign FFPE sample, LNCaP cell line (ATCC), human normal prostate RNA (Catalogue AM7988, ThermoFisher, Waltham, MA, USA) and qPCR human reference total RNA (Catalogue 636690, Clontech, Mountain View, CA, USA), for relative expression determination and normalization across plates. Endogenous control genes ACTB, HPRT1, MRFAP1 and SLC25A3 were tested. qPCR data were analyzed using the ΔΔ Ct method using the lowest expressing, least variable genes (MRFAP1; SLC25A3). We found highly expressed endogenous control genes of limited use in reliably detecting and quantifying transcripts with much lower expression levels on the Fluidigm platform.

### 2.13. Quantification and Statistical Analysis

Relative gene expression levels between each sample type were compared and evaluated using either unpaired *t*-tests (parametric data) or Mann–Whitney tests (non-parametric data) in GraphPad Prism v6; statistical significance was assumed at *p*-value < 0.05. 

## 3. Results and Discussion

### 3.1. The Transcriptomic Landscape of Prostate Cancer 

**Figure 3:**
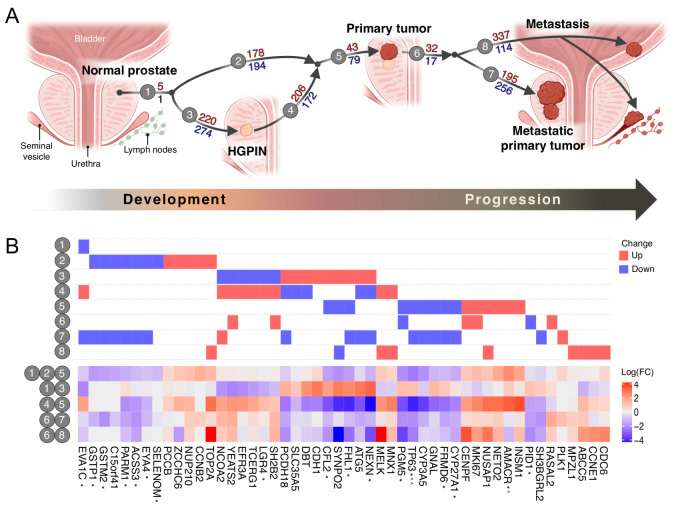
The transcriptomic landscape of prostate cancer development and progression. (**A**) Biological comparisons between tissue types were performed and the top 500 genes were used to assemble the molecular alteration map (MAM) of prostate transcriptional space (Methods). Circled numbers 1–8 indicate individual PC development or progression stages presented in the context of the prostate gland and surrounding organs. The number of up- and down-regulated genes between stages are indicated by red and blue text, respectively. (**B**) Diagram (**top**) and heatmap (**bottom**) illustrating the direction and fold-change (FC), respectively, of expression alterations for selected novel or biologically relevant genes across individual MAM stages (**top**) and biological comparisons (**bottom**). The FC values are presented in log_2_ scale. * in silico prediction validated in a clinical cohort (*p*-value < 0.05); ^ positive control; ^^ negative control.

Several studies have described distinct molecular subtypes of primary PC [6,7], and key genetic alterations underlying its development are well established. However, no unified transcriptomics landscape linking the key molecular events across the different disease stages has been reported. By combining publicly available gene expression datasets from different prostate tissues and across platforms in a comprehensive integrative analysis, we mapped several genes known to be associated with tumor initiation (MYC, AMACR, GSTP1) and progression (TP63, CENPA, PIK3CB, AR, EZH2, and SRD5A1) (Figure S4). We also identified novel gene expression alterations associated with the transition from normal prostate to HGPIN, localized cancer and ultimately metastatic disease (Figure 3). 

Overall, 1488 of 2396 transcriptomic profiles that passed all QC filtering steps were used in the subsequent integrative analysis (Figure 1 and Table S2). For each platform and biological group comparison the expression fold changes (FC) and *p*-values were computed for all genes. These data were combined across different platforms using Stouffer’s method (Figure 2; Methods) to prevent loss of information from genes present on only some platforms. Finally, the expression changes identified in respective PC stages were tracked to construct a molecular alteration map (MAM) of PC (Figure 3A).

Among the top ranked genes, 50 were of particular interest given their biological relevance, novelty or association with other malignancies (Table 1 and Figure 3B). For instance, NUP210 and CCNB2 were up-regulated in primary PC compared to normal prostate tissues, but not when compared to HGPIN samples (MAM 2, Figure 3B). Together with several other cell cycle genes, CCNB2 has recently been found to be over-expressed in a stem-like sub-population of LNCaP cells with reduced dependence on androgen signaling [8], which is more usually associated with castration-resistant PC (CRPC) than early disease development, supporting the idea that there are sub-populations of cells in primary tumors primed for selection following androgen-deprivation therapy [8]. The elevated expression of INSM1 and NETO2, on the other hand, demonstrated a strong association with primary tumor development from both normal prostate and HGPIN lesions, mirroring the expression of AMACR, an established PC risk gene included as a positive control in subsequent experimental validation (MAM 5, Figure 3B).

We also confirmed the over-expression of known cell cycle genes in the development of primary tumor (CCNB2) and the progression to advanced disease (CDC6, MKI67), and identified four members of a prognostic mRNA gene signature [55] that are particularly relevant in PC progression and the development of metastatic disease: NUSAP1, PLK1, CENPF, TOP2A (MAM 6–8, Figure 3B). In agreement with the mechanism of action of docetaxel (microtubule stabilization and mitotic arrest at G2/M), cell cycle genes including CCNB2 have been shown to be downregulated in patients with advanced disease treated with docetaxel and androgen-deprivation therapy [56]. 

A further four genes were found to be consistently upregulated in the progression to metastatic disease—ABCC5, MPZL1, CCNE1, and RASAL2—that were not found in previous, individual studies, despite having clear roles in metastatic progression in other contexts (MAM 6 and 8, Figure 3B). ABCC5 has previously been associated with paclitaxel resistance [29]. Elevated MPZL1 levels have been shown to enhance the migratory and metastatic potential of hepatocellular carcinoma cells [18], while CCNE1 is a marker of poor prognosis in breast, ovarian, and lung cancers, and is frequently over-expressed in tumors, resulting in widespread genomic instability and resistance to trastuzumab in HER2+ breast cancer patients [20]. RASAL2 typically acts as a tumor and metastasis suppressor, except in triple-negative breast cancer (TNBC), where its activation is oncogenic [57]. RASAL2 up-regulation has also been seen in high-grade serous ovarian cancer, which has been found to share a similar molecular portrait to TNBC [58]. Given the shared etiology of these hormone-related cancers, a similar mechanism may be triggered in advanced PC.

Several genes were down-regulated in primary PC compared to benign tissues, and almost all were further down-regulated in the progression to metastatic disease—EVA1C, ACSS3, C15orf41, PARM1, EYA4, GSTM2 and GSTP1 (MAM 1, 2 and 7, Figure 3B). EVA1C has not previously been reported in cancer, but ACSS3 is a marker for gastric cancer [40]. PARM1 is androgen regulated, with a role in cell proliferation [59]. EYA4 has been implicated in several cancers due to its role in DNA double strand break repair and apoptosis [50], while GSTM2 expression reduces oxidative-stress associated inflammation and apoptosis [60]. GSTP1 is involved in the viability and motility of PC cells and GSTP1 hypermethylation has been suggested as a urinary biomarker of PC [61]. Together with EYA4 and GSTM2, GSTP1 undergoes significant epigenetic silencing in primary tumors [30,50] and HGPIN [61], where it may be particularly informative in identifying men suspected of having cancer despite negative biopsies.

CDH1 has also been identified as a prognostic urinary marker of undiagnosed PC in men with HGPIN [10]. Both CDH1 and FHL1 have roles in cell adhesion and migration, and are upregulated in HGPIN compared to benign tissues (MAM 3, Figure 3B). Their loss is associated with progression in several cancers [10,11], and we confirmed the reduction in FHL1 expression from primary to metastatic disease (MAM 5 and 7, Figure 3B). Interestingly, CDH1, FHL1, and GSTP1 have been identified as genetic drivers of an epigenetic field of cancerization in apparently histologically normal tissues in several solid tumors, including prostate [11,32]. Their identification here, and the usefulness of GSTP1 and CDH1 as prognostic markers of PC in some men with negative biopsies [10,61], appears to confirm the significance of the tumor microenvironment as an important factor in prostate tumorigenesis even after filtering the stroma-associated signal (Methods). 

Other genes we identified as down-regulated in the progression from primary cancer to more aggressive disease include FRMD6, CYP3A5, GNAL, PGM5, SH3BGRL2, PID1, CYP27A1, and TP63—an established PC risk gene included as a negative control in the subsequent experimental validation (MAM 5–7, Figure 3B). CYP3A5 facilitates the translocation of the androgen receptor into the nucleus, and, together with CYP27A1 (hypermethylated across all TCGA PC subtypes), has been suggested as a therapeutic target for anti-androgen therapies [38]. GNAL and PGM5 are novel candidates in PC.

### 3.2. In Silico Predictions Validate in Clinical Samples 

Of the top 500 genes predicted to be significantly dysregulated in the transcriptomic landscape of PC by our analysis, we selected 50 genes for further testing by qPCR based on their biological function, novelty or reports of association with other malignancies (Table 1). Gene expression targets were tested in 38 patients with 59 macro-dissected normal/tumor (N/T) formalin-fixed paraffin-embedded (FFPE) tissues and 29 patients with 87 macro-dissected N/HGPIN/T FFPE tissues (Methods; Table S3). We validated 18 genes in this way (*p*-value < 0.05), including 9 genes not previously reported in PC (Table 1): NEXN, PGM5, FRMD6, PARM1, PID1, ACSS3, EVA1C, EYA4 and SELENOM.

**Figure 4:**
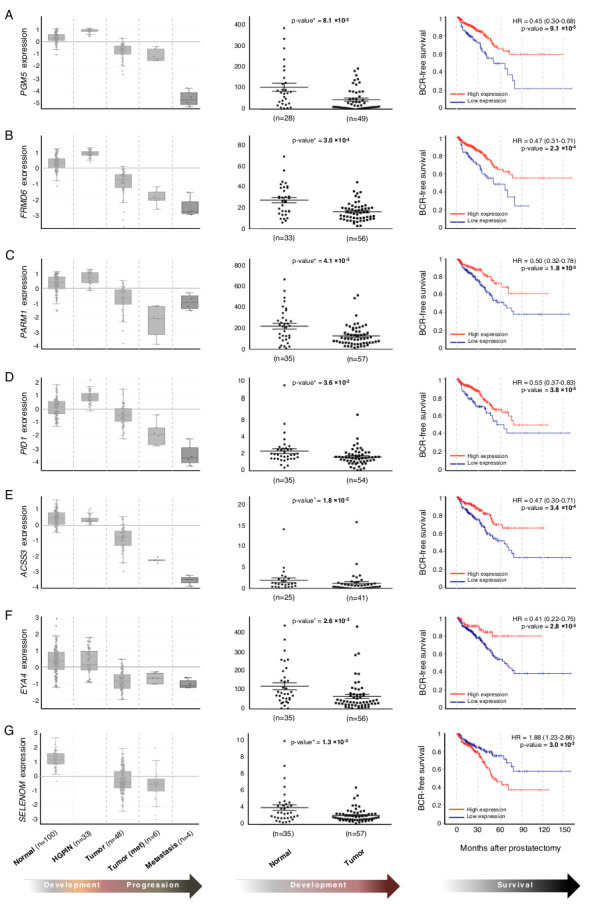
Genes identified by the integrative analysis validate in a clinical cohort. Predicted in silico expression patterns across PC development and progression stages for 7 experimentally validated genes (left panels), including (**A**) PGM5, (**B**) FRMD6, (**C**) PARM1, (**D**) PID1, (**E**) ACSS3, (**F**) EVA4 and (**G**) SELENOM, which were validated in the clinical cohort (middle panels) and stratified TCGA patients into prognostic groups based on low vs. high expression levels (right panels). The in silico expression profiles are based on combined data from 5 Affymetrix HG U133 Plus 2.0 datasets containing 191 samples representing all biological groups used for PC MAM construction. Only genes previously unreported in PC are shown. ^ Mann-Whitney; * unpaired t-test; HR: hazard ratio; BCR: biochemical relapse.

We next performed survival analyses for each gene, using TCGA data (*n* = 387; Methods): 14 of the 18 validated genes stratified patients into prognostic groups based on low vs. high expression levels, including TP63, and 7 of the 10 novel targets—PGM5, FRMD6, PARM1, PID1, ACSS3, EYA4, and SELENOM (Figure 4). Associated Kaplan–Meier log-rank *p*-values are listed in Table 1. This further supports the identification of these genes as transcriptionally important in the development of PC, and their potential usefulness as prognostic markers.

Our transcriptomic landscape has confirmed several known PC risk genes in pathways involved in various key stages of the development (oxidative stress, inflammation) and progression (EMT, dsDNA break repair, cell cycle control) of the disease. It has also revealed several new candidate genes with biological significance and prognostic potential that add to our understanding of the mechanisms underlying the transitions from normal tissue to HGPIN, localized tumor or metastatic disease.

### 3.3. HGPIN Molecular Changes 

Our integrative analysis identified a number of known and novel gene expression targets relevant in the progression of PC, and the inclusion of a single small study with HGPIN samples (*n* = 33 after stringent QC; the only suitable dataset available) in our in silico analysis allowed us to identify several genes with previously undescribed reversible expression patterns in HGPIN tissue, compared to benign or primary PC (e.g., LGR4, TCERG1, SLC35A5, DBT, CFL2, EFR3A; MAM 3 and 4/5; Figure 3B, Table 1). Expression levels of these genes were initially elevated or reduced in HGPIN compared to benign, and subsequently decreased or increased in the progression to primary tumor. In some cases, further alterations in gene expression were associated with progression to aggressive disease (e.g., FHL1, NEXN, SYNPO2, SH2B2, ATG5, NCOA2, YEATS2; MAM 3 and 4, and 6–8; Figure 3B). Overall, 8 of the 13 genes identified are new candidates in PC (Table 1), and warrant functional follow-up study as potential biomarkers, particularly in the context of HGPIN. A total of five have roles in cellular proliferation (SH2B2), adhesion or migration (NEXN, FHL1, SYNPO2) or epithelial to mesenchymal transition (LGR4) and have been associated with progression to aggressive disease in various cancers [62]. Significantly, in addition to contributing to an epigenetic field effect (with CDH1 and GSTP1), Src-mediated phosphorylation of X-linked FHL1 tumor suppressor has recently been reported to convert this gene to a tumor promoter, even when total expression is reduced [63]. This may help explain the apparently ‘reversible’ mRNA expression patterns observed in HGPIN compared to benign or primary tumor tissues, with altered FHL1 contributing at least in part to these changes. However, only SYNPO2 subsequently validated in a comparably sized (*n* = 29) clinical cohort with matched tumor, normal and HGPIN tissues (Figure S5), while FHL1, NEXN, LGR4, and CFL2 showed differential expression only between T vs. N. 

The significant technical challenges involved in accurately identifying, isolating and analyzing HGPIN samples are neatly summarized by Haffner and Barbieri (2016) [64], and we are therefore wary of over-interpreting these data. Additional HGPIN transcriptomic datasets are needed to validate these early intriguing findings, and elucidate the underlying molecular mechanisms and their clinical implications, given the recently reported observation of clonal expansions of cells into histologically-normal tissue [65] and the emerging shift in clinical importance of HGPIN [64].

### 3.4. Pathways Involved in Prostate Cancer Development and Progression are Distinct 

The analysis of gene function generally provides a better description of tumor biology than an analysis of each individual differentially expressed gene [66]. So, in addition to the biological roles of particular genes identified (Table 1), adding to the gene expression and survival analysis data, we also applied Ingenuity Pathway Analysis (IPA) to the expression changes observed in the assembled MAM to identify and map affected pathways to individual stages of PC development and progression. The 232 significantly deregulated canonical pathways (*p* value < 0.05) across all areas on the MAM were complemented with pathway enrichment results based on KEGG, PANTHER, and Reactome pathway databases. Overall, substantial agreement was observed between the resources: there were 34 of 58 (59%), 13 of 27 (48%), and 14 of 19 (74%) significantly enriched pathways in common between IPA and KEGG, PANTHER, and Reactome, respectively (Supplementary Figure S6A). In this way, we confirmed several common biological pathways known to be essential in the development, progression and maintenance of the disease, including androgen signaling, cell cycle and checkpoint regulation, DNA damage control, and protein biosynthesis, and mapped them to the MAM (Figure S6B).

We compared our findings to the ‘molecular concepts’ identified by Tomlins et al. (2007) [67] in 22 benign, 13 HGPIN, 30 primary tumor, and 20 metastatic laser captured micro-dissected samples. We found substantial overlap with their progression model, with androgen signaling predominantly enriched at the HGPIN stage and the cell cycle-related pathways correlating with disease progression in both our studies (Figure S6B). However, whereas we found protein biosynthesis to be correlated with HGPIN phenotype, Tomlins et al. found it associated with both HGPIN and metastatic disease. The inverse enrichment levels observed between androgen receptor (AR) signaling and cell cycle-related pathways between primary and advanced disease also agree with the inverse association of AR activity with cell proliferation reported by [68] in 176 primary and metastatic tumors, and confirmed in the Grasso et al. (2012) [69] dataset (*n* = 61 metastatic or localized PC). 

We also found substantial overlap (35 of 39) between the pathways identified in our study and those identified in a meta-analysis of 18 array datasets, where IPA was used to identify canonical pathways involved in the transition from normal prostate to localized and metastatic disease [70]. Interestingly, many pathways involved in the transition from normal prostate to primary tumor by Gorlov et al. [70] were associated with HGPIN in our study. This could be due to the limited analyses performed, as gene expression changes associated with HGPIN were not explored at the pathway level in that study. Notably, the significant enrichment of integrin signaling in our dataset (Figure S6) is consistent with their key finding that dysregulation of integrin-based cell adhesion is essential in PC progression, which formed the basis of their ‘collagen hypothesis’ of prostate tumorigenesis. Indeed, this is one of only two deregulated pathways we found to be significantly enriched by all resources, and predominates in the transition from localized to metastatic disease (MAM 6–8), which accords with the increased disorganization and motility of cells as they detach from the extra-cellular matrix with an age-associated reduction in integrin ligands like collagen. This is further supported by our identification of deregulated actin cytoskeleton signaling as enriched in the development of PC (MAM 1-3-4-5, *p* = 0.02; data not shown), the biological function most frequently associated with clinically relevant traits [66], as well as multiple genes with roles in cell adhesion, migration, and invasion (Table 1).

**Figure 5:**
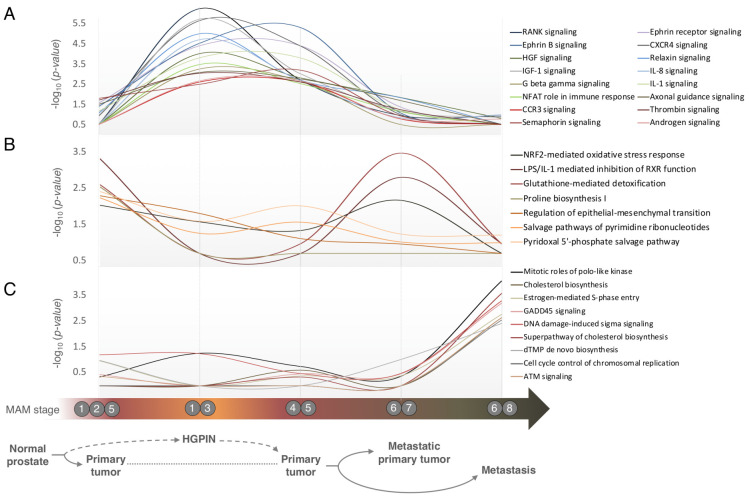
Pathways enriched at distinct stages of disease development and progression. Enrichment patterns across PC development and progression stages for selected pathways significantly associated with (**A**) HGPIN, (**B**) primary and (**C**) metastatic disease (*p*-value < 0.01). Corresponding MAM stages (x-axis) are denoted by circled numbers under the plots. The pathways enrichment is based on IPA *p*-values (Fisher’s exact test) presented in -log_10_ scale (y-axis).

Insulin receptor (IR) signaling was also enriched across all resources (Figure S6A), with the closely related insulin-like growth factor 1 (IGF1) signaling pathway significantly enriched in HGPIN (MAM 1–3, Figure 5A). Binding of their respective ligands activates distinct downstream pathways controlling glucose, lipid and protein metabolism (IR), and proliferation (IGF1), reflected in the MAM. IR and IGF1 signaling pathways are deregulated in Type 2 Diabetes (T2D) and targeted by metformin, which also suppresses androgen signaling pathways that sustain proliferation of normal and tumor prostate cells. However, the anti-neoplastic effects of metformin in PC are not straightforward, especially in the presence of obesity as a confounder, where it appears that metformin can nullify the apparently protective effect of T2D against PC, at least in the early stages of tumor development (reviewed in [71]). Indeed, insulin receptor signaling appears to track androgen signaling in our analysis and are both particularly enriched in the early stages of PC (MAM 1-3-4-5 Supplementary Figure S6B), suggesting that metformin use at these stages in men without T2D may be an effective treatment period. Clinical trials like the METAL study (Metformin and longevity) [72] are currently underway to clarify the mechanism of action of metformin on localized PC, and the optimal stage of disease development to target this drug. While our findings broadly agree with earlier meta-analyses [70], those original data were from limited numbers of samples profiled mainly with low-coverage microarray platforms and representing only certain stages of the disease, so our integrative approach provides a more comprehensive overview of pathways across all stages of PC. Based solely on *p* values ≤ 0.01, the greatest number of deregulated canonical pathways were found in the transition from benign to HGPIN (88/229) and from HGPIN to primary tumor (28/229) (Figure 5 and Figure S7), which agrees with our understanding of the vast cascade of events required to push a cell towards tumorigenesis, absent the activation of key oncogenes

This broad insight into PC transcriptional architecture also revealed distinct enrichment patterns of pathways at particular disease stages. For instance, our analysis revealed the significant enrichment of multiple pathways related to axon guidance (RANK, semaphorin, ephrin B, ephrin receptor, HGF, and axonal guidance signaling) in the progression to primary tumor via HGPIN (Figure 5A), which is a key process in the formation of the neuronal network. Associated genes also affect cellular proliferation, invasion, and angiogenesis [73], supporting its involvement in early neoplastic transformation and tumor progression. At the gene level, EVA1C, CFL2 and NUP210 were identified, which may be particularly relevant given their apparent up-regulation in the development of HGPIN and primary tumor from benign tissues (MAM 2–4, Figure 3B; Table 1). Pathways relating to inflammation (which supports multiple hallmarks of cancer) were also enriched in this transition (CCR3 signaling in eosinophils, NFAT in regulation of the immune response, CXCR4, IL-1, IL-8, thrombin, relaxin and G beta gamma signaling). Furthermore, several genes we identified are involved in the many coordinated biological processes that comprise the immune response. Insulin-like growth factor 1 (IGF1) signaling was also seen here, which has a central role in the progression of many tumors including PC, via activation of the PI3K/AKT pathway. Elevated IGF1 signaling has also been linked with androgen independence and as such suggested as a drug target in CRPC (reviewed by [74]). More generally however, HGPIN appeared to lack abnormal metabolism pathways and is under-represented for oxygen and other stress-related cellular response pathways compared to primary or metastatic disease. 

In contrast, far fewer pathways were enriched in the ‘direct’ development of cancer from benign tissue (MAM 1-2-5), i.e., not via HGPIN (7 vs. 21; Figure 5B), and included functions relating to specific metabolic alterations (pyridoxal 5’-phosphate (vitamin B6) salvage, glutathione-mediated detoxification, proline biosynthesis, salvage pathways of pyrimidine ribonucleotides). Proline synthesis has been observed as a major metabolic shift in several cancer models, and has a critical role in promoting tumor cell growth [75]. Furthermore, proline can be derived from collagen degradation [76], and proline biosynthesis was uniquely enriched in this transition, where regulation of the epithelial-to-mesenchymal transition pathway was also observed. Together, these further support the collagen hypothesis of PC development [70]. 

Overall, three pathways broadly relating to cellular damage by reactive oxygen species (ROS) and exogenous compounds were enriched in both the development of primary tumor (MAM 1-2-5) and in the progression to aggressive disease (MAM 6–7, Figure 5B). The LPS/IL-1 mediated inhibition of Retinoid X Receptor (RXR) is associated with impaired metabolism of cholesterol and xenobiotics [77]. RXR/LXR activation pathways were also found to be significantly downregulated in a recent urinary proteomic analysis [78]. The NRF2-mediated oxidative stress response pathway activates cytoprotective antioxidants and detoxifying enzymes, but hyperactivation of NRF2 is associated with chemoresistance, due to its rapid action preventing the necessary accumulation of drugs and inhibiting the desired treatment-associated apoptotic response [79]. Glutathione-mediated detoxification of ROS and xenobiotics is central in maintaining cellular redox balance and the elimination of carcinogens. However, elevated levels of glutathione are also associated with tumor phenotype and, with increased levels of glutathione-S transferases (GST), can contribute to chemoresistance, by attenuating the desired effects of a given drug [80] by mechanisms broadly similar to those described above. At the gene level, our analysis identified numerous targets involved in these processes (GSTP1, GSTM2, CYP3A5, TCERG1, ATG5, ABCC5, SLC35A5, ZCCHC6, MPZL1, NETO2, and PID1), three of which validated (T vs. N) in a clinical cohort, and which were also prognostic in TCGA data: GSTM2, GSTP1, PID1 (Table 1). 

Overall, three main biological processes were identified in the progression to metastatic disease: cell cycle control, DNA damage response and cholesterol biosynthesis (MAM 6–8, Figure 5C). The latter is consistent with the idea that cholesterol is part of the ‘cell economy’ and a vital aspect of cell proliferation and growth [81]. Elevated cholesterol levels are associated with PC progression [82], and a precursor of intra-tumoral de novo androgen synthesis observed in the progression to castration-resistant PC [83]. At the gene level, ACSS3 and CYP27A1 were both found to be downregulated in the development of primary tumor (MAM 2, 5) and in the development of aggressive disease (MAM 5–7). Both targets validated (T vs. N; *p*-value = 0.018 and 0.011, respectively) in the clinical cohort and were also prognostic in TCGA data (Figure 4 and Table 1). ACSS3 activates acetate for use in lipid synthesis, while CYP27A1 encodes an enzyme involved in regulating cellular cholesterol homeostasis. It is dramatically downregulated in PC, and associated with higher tumor grade and reduced disease-free survival [42]. 

Deregulated cell cycle control is an established feature of tumor development, exemplified in our analysis with the enrichment of pathways relation to de novo dTMP synthesis, cell cycle control of chromosomal replication, estrogen-mediated S-phase entry and mitotic roles of polo-like kinase (MAM 6–8, Figure 5C), as well as specific genes (Table 1) Closely related pathways relating to DNA integrity were also over-represented, including G2/M DNA damage checkpoint regulation, DNA damage sensor ATM signaling, its downstream target GADD45 signaling, and the tumor suppressor DNA damage-induced 14-3-3 sigma signaling pathway. Notably, mutations and deletions in DNA repair genes affected almost 20% of the TCGA cohort. Specifically, ATM was recurrently mutated in 6% of all primary tumors and across all TCGA subtypes, emphasizing the broad clinical significance of DNA damage repair in PC. At the gene level, we identified and validated down-regulated EYA4 as a novel target in the development of aggressive disease (T vs. N, *p* = 0.026) and also found it to be prognostic in TCGA data (log-rank *p*-value = 2.8 × 10^−3^) (Figure 4).

## 4. Conclusions

In summary, this is the largest multi-cohort study of PC gene expression to date and provides a comprehensive overview of the transcriptomic landscape of PC. We established an alternative analytic approach that bypasses the current limitations of available tools and supports the comprehensive integration of data derived from different microarray and sequencing platforms and disease subtypes to connect distinct disease stages and reveal potentially relevant genes not identifiable from single studies alone. The application of our methodology to the combined total of TCGA data and several other high-quality studies into different PC stages and grades facilitated the reconstruction of the molecular history of this disease.

Our identification of known risk genes and biological pathways known to be relevant in the development of PC demonstrates the reliability of our cross-platform data integration approach to identify novel targets supported by qPCR validation of specific gene targets, associated survival analysis of TCGA dataset, and disease-stage specific pathway analysis using IPA, KEGG, Reactome, and Panther. The ability to identify genes associated with particular stages of disease development or progression provides a resource for novel biomarker development that we believe will help to understand underlying disease mechanisms. By adopting this global approach, pathways relating to both integrin and insulin signaling have emerged as particularly relevant in the global transcriptomic landscape of PC, supporting earlier suggested fundamental disease mechanisms (collagen hypothesis; insulin signaling). To allow others to explore the assembled data, we also developed a user-friendly integrative expression database. We hope these findings and associated online tool will enhance translational research and inform patient management. 

The collected and integrated data along with analytical and visualization tools (e.g., PCA, gene expression analyses or heatmaps and Pearson correlations, gene networks, survival analyses) are freely available online through our Prostate Integrative Expression Database at www.pixdb.org.uk.

## Figures and Tables

**Table 1:** Genes associated with prostate cancer or development.

Gene	Sample Ns (T,HGPIN,N)	Validation *p*-Value	Confirms in Silico Results?	Log-Rank Test *p*-Value (TCGA)	Biological Role	Association with other Malignancies	Reference
*AMACR* ^a^	55, 0, 36	1.9 × 10^−2^	Yes (T vs. N)	6.3 × 10^−2^	Racemase processing bile for degradation	Luminal cell marker in PC	[9]
*TP63* ^b^	22, 23, 22	6.9 × 10^−3^	Yes (T vs. N)	1.3 × 10^−3^	Tumor suppressor	Basal cell marker in PC	[9]
*CDH1* ^c^	11, 12, 7	0.26	-	1.5 × 10^−4^	Cell adhesion	Marker of PC in patients with HGPIN	[10]
*FHL1* ^c,f^	40, 0, 25	1.1 × 10^−3^	Yes (T vs. N)	4.7 × 10^−2^	Cell adhesion, migration, colony formation	Forms epigenetic field defect in GISTs	[11]
*PCDH18* ^d^	51, 0, 26	0.3	-	-	Cell adhesion, migration, colony formation	Hypermethylated in CRC	[12]
*NEXN* ^d,f^	55, 0, 35	7.3 × 10^−3^	Yes (T vs. N)	8.3 × 10^−2^	Cell adhesion, migration	Cardiomyopathy	[13]
*SYNPO2* ^f^	23, 22, 24	1.2 × 10^−2^	Yes (T vs. N)	5.5 × 10^−4^	Cell migration	Invasive cancer biomarker	[14]
*PGM5* ^d^	49, 0, 28	8.1 × 10^−3^	Yes (T vs. N)	9.1 × 10^−5^	Adherens-type cellular junctions	Downregulated in CRC	[15]
*RASAL2* ^d^	21, 20, 18	0.5	-	-	RHO-GAP; cell invasion	Key roles in tumor progression and metastasis	[16]
*LGR4* ^f^	55, 0, 35	3.4 × 10^−3^	Yes (T vs. N)	1.7 × 10^−1^	Activates Wnt signaling	Key protein in PC metastasis	[17]
*SH2B2* ^d,f^	21, 15, 14	0.63	-	-	Cellular proliferation		
*FRMD6* ^d^	56, 0, 33	3 × 10^−4^	Yes (T vs. N)	2.3 × 10^−4^	Cellular proliferation	Tumor suppressor in BrCa	[18]
*PARM1* ^d^	57, 0, 35	4.1 × 10^−3^	Yes (T vs. N)	1.8 × 10^−3^	Cellular proliferation	Oncogenic in leukemias	[19]
*CCNE1* ^d^	10, 12, 14	0.65	-	-	Cell cycle progression	Chromosomal instability and trastuzumab resistance; poor prognosis in multiple cancers	[20,21]
*INSM1* ^d^	24, 24, 22	0.61	-	-	Cell cycle progression	Regulates NE differentiation in several tumor types	[22]
*CCNB2*	8, 3, 9	0.79	-	-	Cell cycle progression	Recurrent PC	[8]
*MKI67*	26, 0, 7	5.1 × 10^−3^	-	-	Cell cycle progression	Recurrent PC	[8]
*MELK*	49, 0, 29	0.14	-	3.5 × 10^−6^	Cell cycle progression	High-grade PC	[23]
*NUSAP1* ^e^	45, 0, 22	0.83	-	-	Cell cycle progression	Promotes invasion and metastasis PC	[24]
*PLK1* ^e^	11, 13, 10	0.8	-	-	Cell cycle progression	Recurrent PC	[8]
*CENPF* ^e^	54, 0, 29	0.56	-	3.3 × 10^−9^	Cell cycle progression	Recurrent PC	[8]
*TOP2A* ^e^	45, 0, 21	0.46	-	-	Cell cycle progression	Marker of aggressive PC	[25]
*MYC*	14, 15, 10	0.39	-	-	Cell cycle progression; apoptosis	Upregulated in HGPIN and PC	[26]
*TCERG1* ^d,f^	7, 8, 6	0.22	-	-	Transcriptional elongation and splicing; lipid homeostasis (*C.elegans*)	Sensitizes cell to apoptosis	[27]
*ATG5* ^f^	19, 23, 21	0.63	-	-	Apoptosis; autophagy	Increased levels in NE PC	[28]
*ABCC5* ^d^	25, 25, 26	0.75	-	1.9 × 10^−8^	Cellular export of cyclic nucleotides	Paclitaxel resistance in nasopharyngeal cancer	[29]
*GSTM2*	57, 0, 35	1.8 × 10^−3^	Yes (T vs. N)	2.9 × 10^−2^	Detoxification of electrophilic compounds	Prognostic marker in PC	[30]
*SLC35A5* ^d,f^	19, 19, 18	0.77	-	-	Transmembrane protein	SNPs associated with paclitaxel sensitivity	[31]
*GSTP1* ^c^	56, 0, 33	6 × 10^−4^	Yes (T vs. N)	1.2 × 10^−2^	Drug metabolism; cell cycle regulation	Hypermethylated in PC	[32]
*ZCCHC6* ^d^	24, 23, 21	0.67	-	-	Uridylation of mRNA	Loss (*C.elegans* homologue) leads to chromosomal instability	[33]
*MPZL1* ^d^	23, 23, 23	0.69	-	-	Cell signaling via c-Src	Amplification promotes cell migration in HCC	[34]
*NETO2* ^d^	18, 17, 10	0.41	-	-	Glutamate signaling in neurons	Prognostic in CRC	[35]
*PID1* ^d^	54, 0, 34	3.6 × 10^−2^	Yes (T vs. N)	3.8 × 10^−3^	Insulin signaling	Tumor suppressor in brain tumors and gliomas	[36]
*NCOA2* ^f^	21, 23, 21	0.47	-	-	Transcriptional coactivator of nuclear hormone receptors	AR co-repressor with antiandrogens	[37]
*CYP3A5*	14, 0, 13	0.4	-	-	Nuclear translocation of AR	Regulates growth PC	[38]
*EZH2*	40, 0, 20	0.81	-	-	Transcriptional regulator	Marker of aggressive PC	[25]
*ACSS3* ^d^	41, 0, 25	1.8 × 10^−2^	Yes (T vs. N)	3.4 × 10^−4^	Cholesterogenesis	Prognostic marker in neuroblastoma, gastric cancer	[39,40]
*MNX1*	33, 0, 13	0.33	-	-	Lipid synthesis	Upregulated in African American PC	[41]
*CYP27A1*	54, 0, 30	1.1 × 10^−2^	Yes (T vs. N)	5.6 × 10^−5^	Cellular cholesterol homeostasis	Associated with poor prognosis in PC	[42]
*YEATS2* ^d,f^	44, 0, 24	0.29	-	-	Cellular metabolism; epigenetic regulation	Mutated in multiple tumor types	[43]
*DBT* ^d,f^	43, 0, 23	0.77	-	-	Branched-chain amino acid metabolism	Mutated in maple syrup urine disease	[44]
*PCCB* ^d^	56, 0, 32	0.77	-	-	Catabolism of propionyl-CoA	Reduced expression in CRC	[45]
*NUP210* ^d^	53, 0, 35	0.2	-	7.6 × 10^−3^	Muscle and neuronal differentiation	Upregulated in cervical cancer	[46]
*EVA1C* ^d^	53, 0, 28	1.3 × 10^−2^	Yes (T vs. N)	7.6 × 10^−2^	Axon guidance (mouse)		[47]
*CFL2* ^d, f^	21, 21, 18	5.1 × 10^−2^	Yes (T vs. N)	1.5 × 10^−2^	Axon guidance	Associated with progression in gastric cancer	[48]
*CDC6*	9, 16, 10	0.26	-	-	DNA damage repair	Elevated in progression to PC	[49]
*EYA4* ^d^	56, 0, 35	2.6 × 10^−3^	Yes (T vs. N)	2.8 × 10^−3^	DNA damage repair	Hypermethylated in several cancers	[50]
*GNAL* ^d^	24, 0, 23	0.65	-	-	Odorant signaling	Mutated in HCC	[51]
*EFR3A* ^d,f^	54, 0, 36	0.92	-	-	Responsiveness of GPCRs	SNPs associated with CRC	[52]
*SH3BGRL2* ^d^	31, 0, 54	0.34	-	-	Unknown		
*C15orf41* ^d^	28, 0, 52	0.17	-	-	Unknown	Congenital dyserythropoietic anemia	[53]
*SELENOM* ^d^	35, 0, 57	1.3 × 10^−2^	Yes (T vs. N)	3.0 × 10^−3^	Unknown	IHC marker of aggressive HCC	[54]

^a^ positive control, ^b^ negative control, ^c^ driver of epigenetic field of cancerization, ^d^ not previously reported in PC, ^e^ cell cycle progression signature (Cuzick et al., 2011), ^f^ reversible expression in HGPIN. Abbreviations: GIST: gastrointestinal stromal tumor; CRC: colorectal cancer; BrCa: breast cancer; NE: neuroendocrine; HCC: hepatocellular carcinoma; AR: androgen receptor.

## Data Availability

Requests for further information, access to processed data files or laboratory reagents used can be directed to corresponding author Helen Ross-Adams (h.ross-adams@qmul.ac.uk), at the Centre for Biomarkers and Biotherapeutics, Barts Cancer Institute, Charterhouse Square, Queen Mary University of London, EC1M 6BQ, LONDON, UK. Publicly available datasets supporting this work are listed in Supplementary Table S1. The processed datasets used in the development of this project are available for interrogation, analysis and visualization using Prostate Integrative Expression Database (PIXdb), at www.pixdb.org.uk.

## Supplementary Materials

The following are available online at https://www.mdpi.com/2072-6694/13/2/345/s1, Figure S1: Microarray and NGS processing pipeline, Figure S2: Sample and tissue type quality control, Figure S3: Cross-platform integrative analysis model optimization, Figure S4: The transcriptomic landscape: known genes, Figure S5: Reversible gene expression, Figure S6: Enriched pathways, Figure S7: Pathways enriched at distinct disease stages, Table S1: Datasets used, Table S2: Samples filtering steps and results, Table S3: Gene expression assays.
