# Supplementary material for: The Transcriptomic Landscape of Prostate Cancer Development and Progression: An Integrative Analysis
Source: Cancers (Basel). 2021 Jan 19;13(2):345. doi: 10.3390/cancers13020345 (PMC7838904; doi:10.3390/cancers13020345)
Supplement: Supplementary file 1 [file cancers-13-00345-s001.pdf]

cancers

MDPI

Supplementary materials

The Transcriptomic Landscape of Prostate Cancer Development and Progression: An Integrative Analysis

Jacek Marzec [1,†], Helen Ross-Adams [1,†,*], Stefano Pirrò [1], Jun Wang [1], Yanan Zhu [2], Xueying Mao [2], Emanuela Gadaleta [1], Amar S. Ahmad [3], Bernard V. North [3], Solène-Florence Kammerer-Jacquet [2], Elzbieta Stankiewicz [2], Sakunthala C. Kudahetti [2], Luis Beltran [4], Guoping Ren [5], Daniel M. Berney [2,4], Yong-Jie Lu [2] and Claude Chelala [1,6,*]

[1] Bioinformatics Unit, Centre for Cancer Biomarkers and Biotherapeutics, Barts Cancer Institute, Queen Mary University of London, London EC1M 6BQ, UK; jacek.marzec@unimelb.edu.au (J.M.); s.pirro@ucl.ac.uk (S.P.); j.a.wang@qmul.ac.uk (J.W.); e.gadaleta@qmul.ac.uk (E.G.)

[2] Centre for Cancer Biomarkers and Biotherapeutics, Barts Cancer Institute, Queen Mary University of London, London EC1M 6BQ, UK; yanan.zhu@qmul.ac.uk (Y.Z.); x.mao@qmul.ac.uk (X.M.); soleneflorence.kammerer-jacquet@chu-rennes.fr (S.-F.K.-J.); e.stankiewicz@qmul.ac.uk (E.S.); s.kudahetti@qmul.ac.uk (S.C.K.); d.m.berney@qmul.ac.uk (D.M.B.); y.j.lu@qmul.ac.uk (Y.-J.L.)

[3] Centre for Cancer Prevention, Wolfson Institute of Preventive Medicine, Barts and the London School of Medicine, Queen Mary University of London, London EC1M 6BQ, UK; amar.ahmad@qmul.ac.uk (A.S.A.); b.v.north@qmul.ac.uk (B.V.N.)

[4] Department of Pathology, Barts Health NHS, London, E1 F1R, UK; luis.beltran@bartsandthelondon.nhs.uk

[5] Department of Pathology, The First Affiliated Hospital, Zhejiang University Medical College, Hangzhou 310058, China; 1190024@zju.edu.cn

[6] Centre for Computational Biology, Life Sciences Initiative, Queen Mary University London, London EC1M 6BQ, UK

* Correspondence: h.ross-adams@qmul.ac.uk (H.R.-A.); c.chelala@qmul.ac.uk (C.C.)

† These authors contributed equally

Citation: Marzec, J.; Ross-Adams, H.; Pirro, S.; Wang, J.; Zhu, Y.; Mao, X.; Gadaleta, E.; Ahmad, A.S.; North, B.V.; Kammerer-Jacquet, S.-F.; et al. The transcriptomic landscape of prostate cancer development and progression: an integrative analysis. *Cancers* **2021**, *13*, 345. https://doi.org/10.3390/cancers13020345 10.3390/cancers13020345

Received: 07 January 2021
Accepted: 12 January 2021
Published: 19 January 2021

Publisher's Note: MDPI stays neutral with regard to jurisdictional claims in published maps and institutional affiliations.

Copyright: © 2021 by the authors. Licensee MDPI, Basel, Switzerland. This article is an open access article distributed under the terms and conditions of the Creative Commons Attribution (CC BY) license (http://creativecommons.org/licenses/by/4.0/).

Supplementary

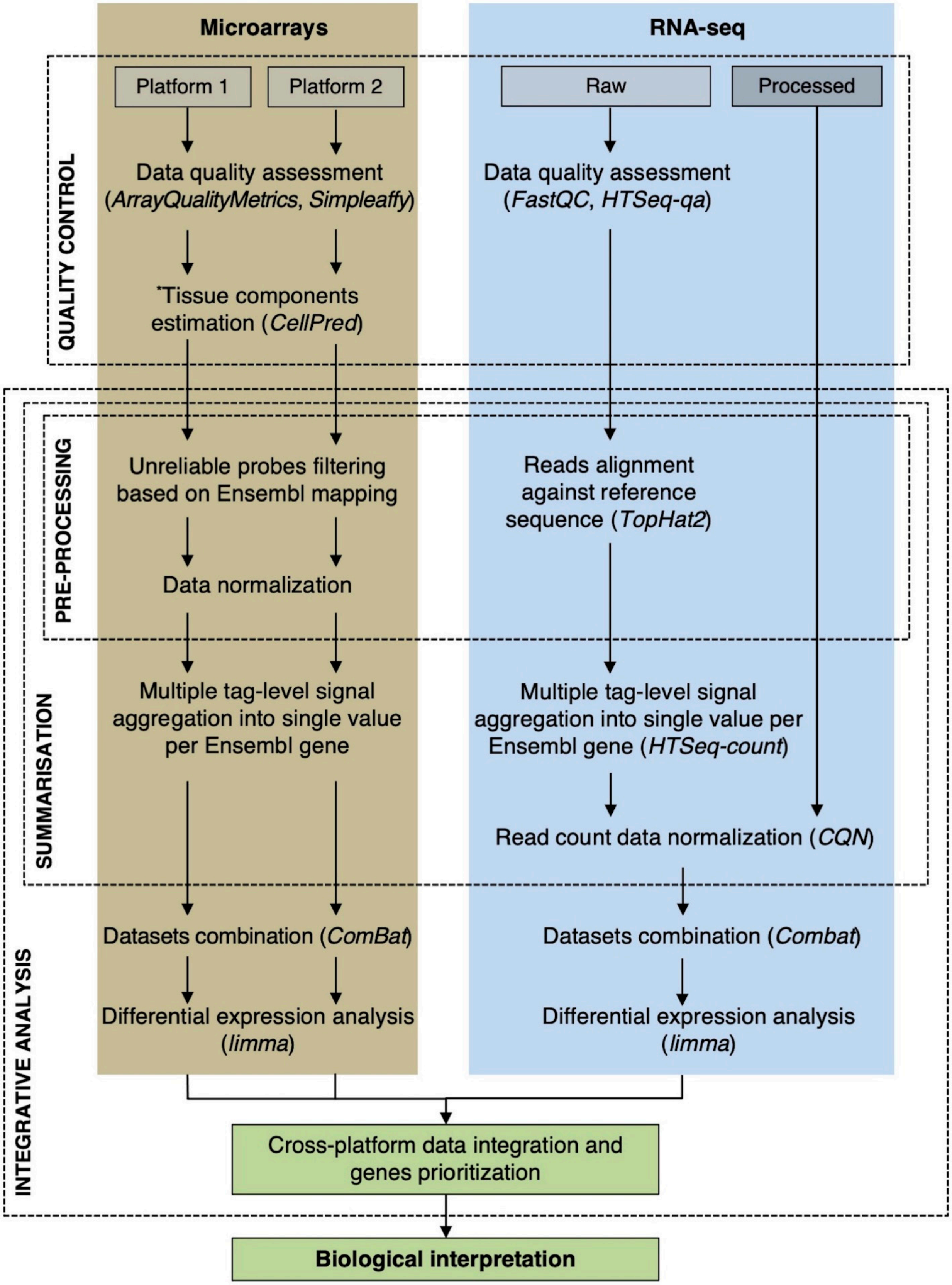

Figure S1. Microarray and NGS data processing pipeline.Data quality control and pre-processing, gene expression aggregation and per-platform differential expression analyses were followed by cross-platform integrative analysis. * Applicable to samples processed using Affymetrix HG U133 Plus 2.0, U133A or U95Av2 array platforms.

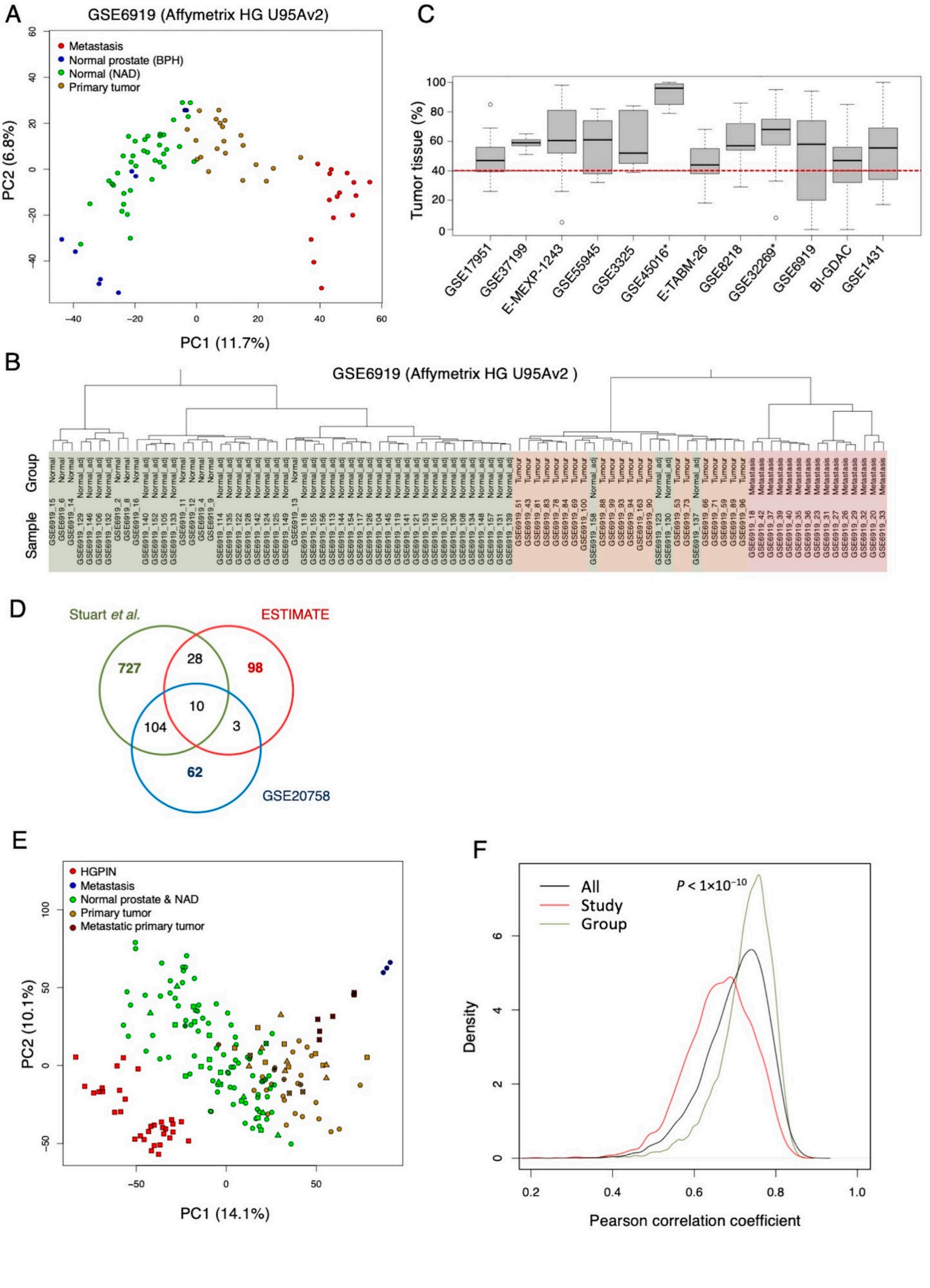
A
GSE6919 (Affymetrix HG U95Av2)
Metastasis
Normal prostate (BPH)
Normal (NAD)
Primary tumor
PC2 (6.8%)
PC1 (11.7%)
C
Tumor tissue (%)
GSE17951
GSE37199
E-MEXP-1243
GSE55945
GSE3325
GSE45016*
E-TABM-26
GSE8218
GSE32269*
GSE6919
BI-GDAC
GSE1431
B
GSE6919 (Affymetrix HG U95Av2)
Group
Sample
Normal
Normal_adj
Tumour
Metastasis
D
Stuart et al.
ESTIMATE
GSE20758
727
28
98
10
104
3
62
E
HGPIN
Metastasis
Normal prostate & NAD
Primary tumor
Metastatic primary tumor
PC2 (10.1%)
PC1 (14.1%)
F
All
Study
Group
P < 1×10−10
Density
Pearson correlation coefficient

Figure S2. Sample and tissue type quality control. **A**. PCA showed clustering of BPH and NAD samples with each other, which were subsequently combined into one 'normal' control set. Data shown are from Chandran et al (2007). **B**. Unsupervised hierarchical clustering identified NAD samples grouping with primary tumors, indicating potential field effect; these were removed from all downstream analyses. Data shown are from Chandran et al (2007) **C**. *in silico* predicted tumor tissue percentage in samples across collected datasets profiled with Affymetrix HG U133 Plus 2.0, U133A and U95Av2 arrays. Only samples with >40% tumor content were retained (dashed red line). * samples obtained by LCM. **D**. Stroma-associated genes from three resources were combined and filtered out before data integration. **E**. Observed variance of the combined and batch effects-adjusted data can be ascribed to sample type (biological effects). **F**. Biological effects were significantly stronger than dataset effects across all datasets; Kolmogorov-Smirnov p-values $<1\times10^{-10}$. Data shown (E, F) are from five Affymetrix HG U133 Plus 2.0 datasets (Table S1) containing 191 samples representing all biological groups used for PC MAM construction.

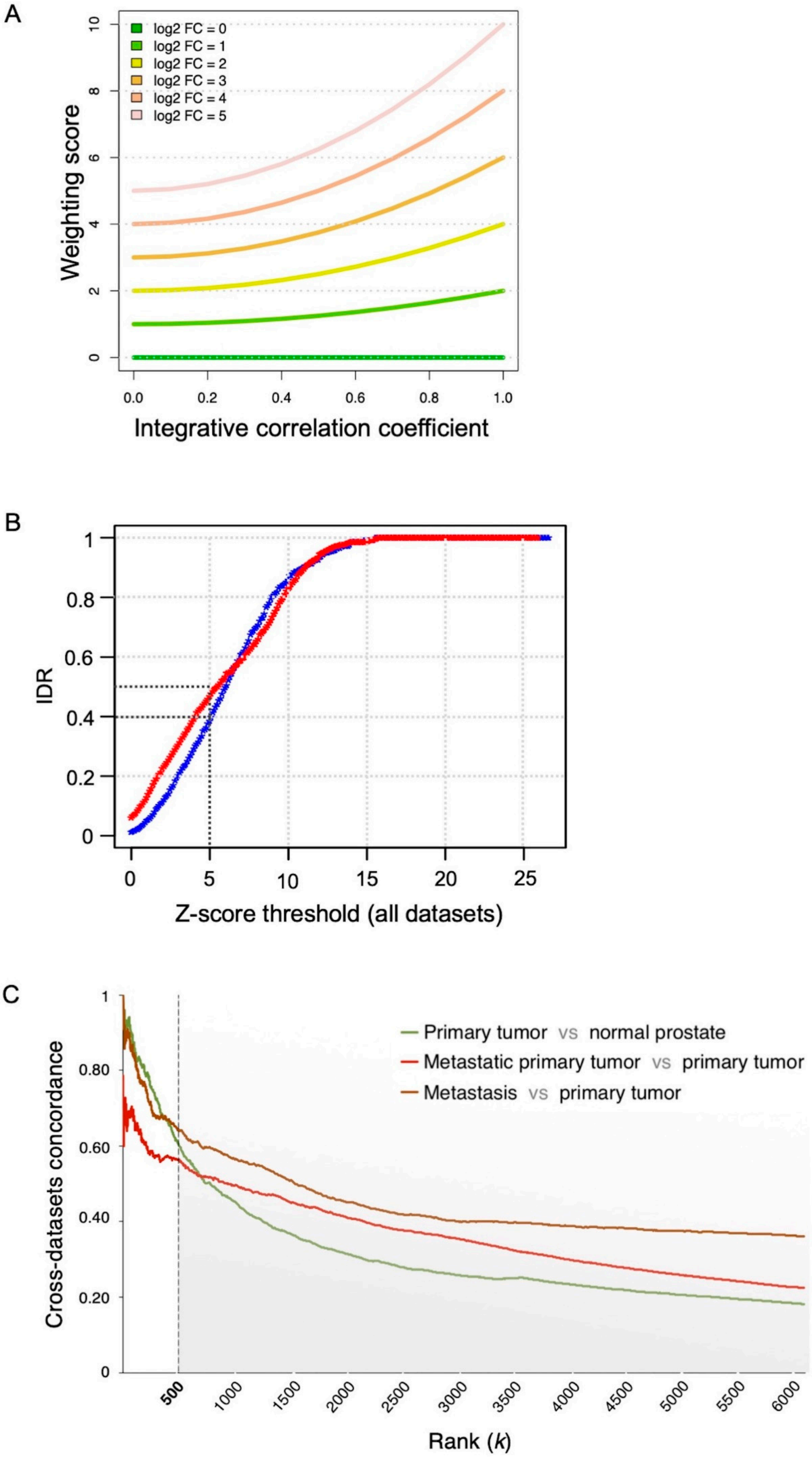
A
log2 FC = 0
log2 FC = 1
log2 FC = 2
log2 FC = 3
log2 FC = 4
log2 FC = 5
Weighting score
0
2
4
6
8
10
0.0
0.2
0.4
0.6
0.8
1.0
Integrative correlation coefficient
B
IDR
0
0.2
0.4
0.6
0.8
1
0
5
10
15
20
25
Z-score threshold (all datasets)
C
Primary tumor vs normal prostate
Metastatic primary tumor vs primary tumor
Metastasis vs primary tumor
Cross-datasets concordance
0
0.20
0.40
0.60
0.80
1
500
1000
1500
2000
2500
3000
3500
4000
4500
5000
5500
6000
Rank (*k*)

Figure S3. Cross-platform integrative analysis model optimization.**A**. Stouffer's method weighting score presented as a function of log_2 fold-change (FC) values and integrative correlation coefficients (ICCs). **B.** Integration-driven discovery rates (IDRs) calculated for primary PC vs normal groups using data derived from the comprehensive platforms. The blue and red lines correspond to Z-scores <0 and >0, respectively. Approximately 40% of all down-regulated and 50% of all up-regulated genes with Z-score > 5 in combined data did not reach this threshold in individual platforms. **C.** Fraction of top-k rankings consistently deregulated across datasets with the top 500 ranked genes used as the threshold in each biological comparison to assemble the molecular alteration map. The log_2FC values in individual biological comparisons were computed for each dataset to assess the fraction (y-axis) of genes with $log_2FC > 1$ (up-regulated) or $log_2FC < -1$ (down-regulated) in at least half of the datasets for cumulative number of top-*k* ranked genes (x-axis).

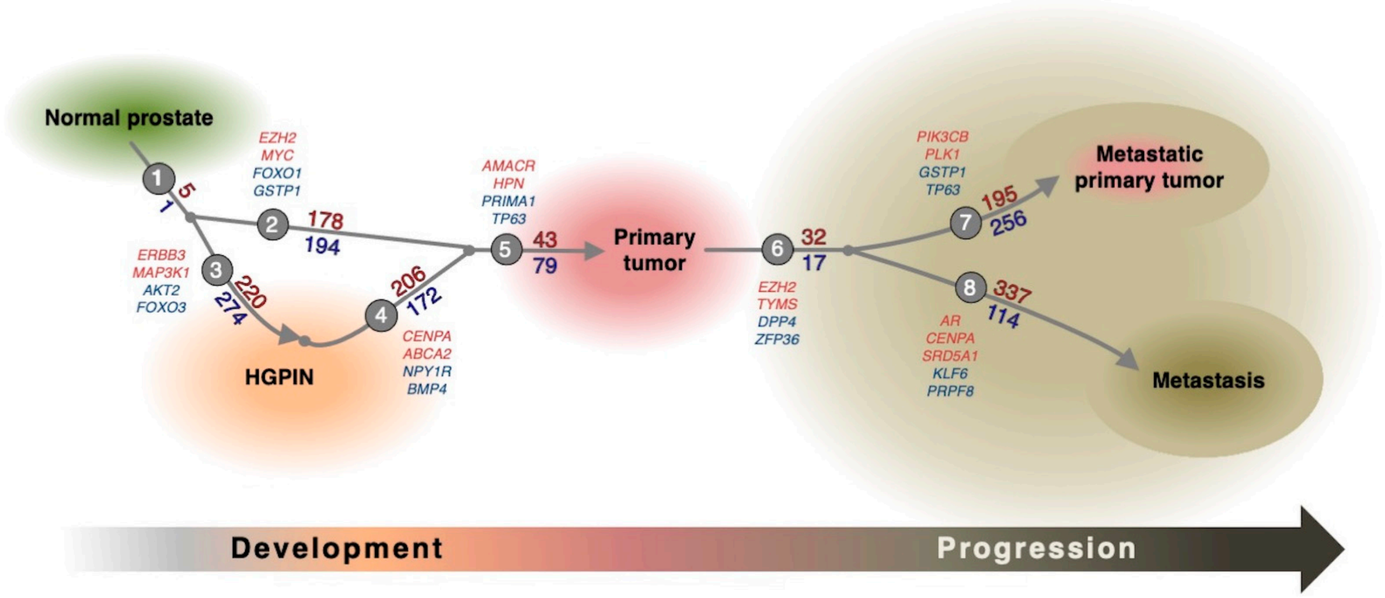

Figure S4. The transcriptomic landscape of prostate cancer development and progression: known genes.Biological comparisons between tissue types were performed and the top 500 genes used to assemble the molecular alteration map (MAM) of prostate transcriptional space (see Methods). Individual PC development or progression stages are indicated by circled numbers 1-8. Selected known PC risk genes identified in the integrative analysis are shown. The names and number of up- and down-regulated genes between stages are indicated by red and blue text, respectively.

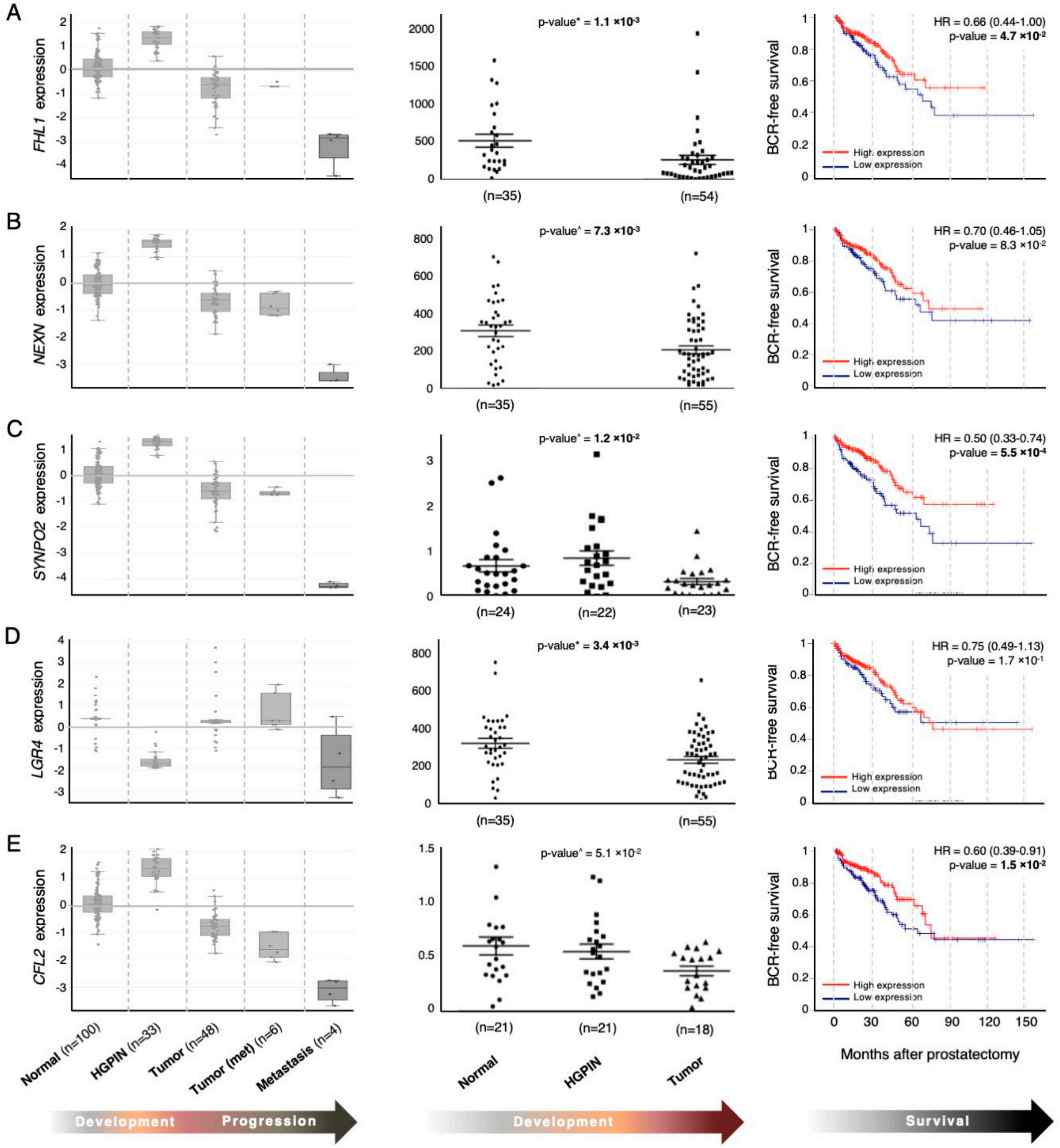

Figure S5. Integrative analysis identified genes with reversible expression in HGPIN. The reversible expression patterns in HGPIN tissue observed *in silico* (left panels) for (**A**) *FHL1*, (**B**) *NEXN*, (**C**) *SYNPO2*, (**D**) *LGR4* and (**E**) *CFL2*, that validated in the clinical cohort (middle panels), with associated survival differences between patient groups based on mRNA expression levels (right panels). The *in silico* expression profiles are based on combined data from five Affymetrix HG U133 Plus 2.0 datasets containing 191 samples representing all biological groups used for PC MAM.

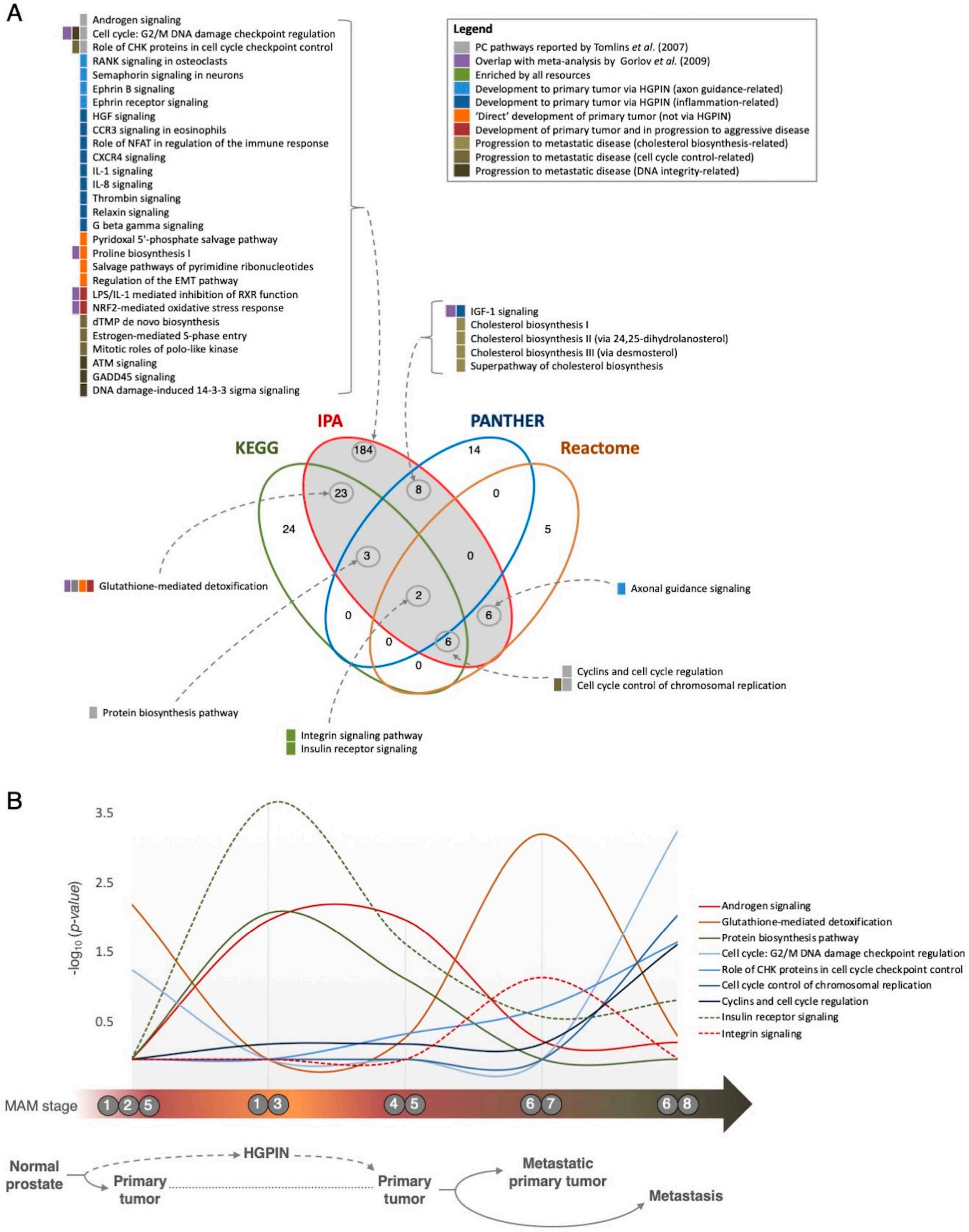

Figure S6. Enriched pathways in the prostate transcriptomic landscape**A.** Overlap of significantly enriched pathways (Fisher's exact test p-value<0.05) according to IPA, KEGG, Panther and

Reactome functional annotation databases. Selected pathways, including molecular concepts from Tomlins et al (2007), are presented. Insulin and integrin receptor signaling pathways were found to be significantly enriched according to all four databases. **B.** Enrichment patterns for significantly enriched pathways that are known to be essential in the development, progression and maintenance of PC, including androgen signaling, glutathione-mediated detoxification, protein biosynthesis and cell cycle-related pathways, together with the insulin and integrin signaling pathways. Corresponding MAM stages are shown on the x-axis. The pathways enrichment is based on IPA p-values (Fisher's exact test) presented in $-\log_{10}$ scale (y-axis).

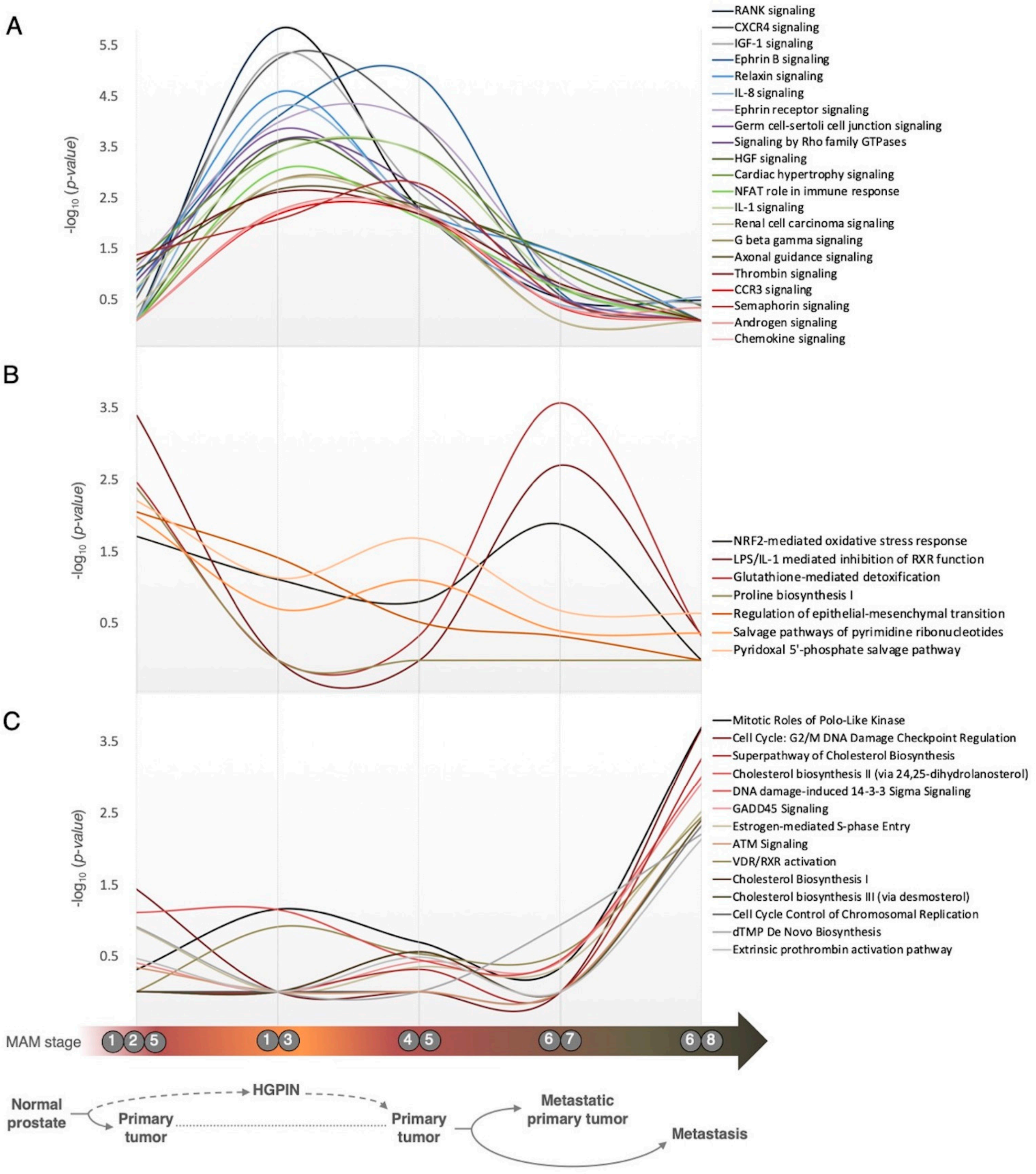

Figure S7. Pathways enriched at distinct stages of disease development and progression.Enrichment patterns across PC development and progression stages for pathways significantly associated with (**A**) HGPIN, (**B**) primary and (**C**) metastatic disease (p-value <0.01). Corresponding MAM stages (x-axis) are denoted by circled numbers under the plots. The pathways enrichment is based on IPA p-values (Fisher's exact test) presented in $-log^{10}$ scale (y-axis).

Table S1. RNAseq and microarray prostate cancer gene expression datasets collated for this study.

Platform			Study	Clinical data	Sample Type	Sample No.	Metastasis site	Excluded (QC)	Reference
**RNA-Seq**	**Illumina**	**HiSeq 2000**	**TCGA**	**Yes**	**Metastatic primary tumor**	**1**	-	0	(Cancer Genome Atlas Research, 2015)
					Primary tumor	497		43	
					Normal (NAD)	52		15	
			ICGC	Yes	Primary tumor	11	-	2	(Weischenfeldt et al., 2013)
					Normal (NAD)	1		0	
		GA II	GSE22260	Yes	Primary tumor	20	-	20	(Kannan et al., 2011)
					Normal (NAD)	10		10	
**MIicroarray**		HumanHT-12 v3	GSE32571	Yes	Primary tumor	59	-	6	(Kuner et al., 2013)
					Normal (NAD)	39		3	
	**Affymetrix**	HuEx 1.0 ST	GSE21034	Yes	Metastatic primary tumor	19	Bone, brain, bladder, colon, lung, neck, node, spine	4	(Taylor et al., 2010)
					Primary tumor	131		26	
					Normal (NAD)	29		16	
			GSE29079	Yes	Primary tumor	47		8	(Brase et al., 2011)
					Normal (NAD)	48		11	
			GSE41408	Yes	Metastatic primary tumor	9	Unknown	0	(Boormans et al., 2013)
					Primary tumor	39		4	
			GSE30521	Yes	Primary tumor	17	-	4	(Agell et al., 2012)
					Normal (NAD)	5		2	
		HG U133 Plus 2.0	GSE32448	Yes	Primary tumor	40	-	40	(Derosa et al., 2012)
					Normal (NAD)	40		40	
			GSE17951	Yes	Primary tumor	30	-	9	(Wang et al., 2010)
					Normal (NAD)	41		3	
					Normal prostate	45		6	
			E-MEXP-1243	Yes	Primary tumor	14	-	6	(Traka et al., 2008)
					HGPIN	49		16	
					Normal prostate	18		6	
			GSE55945	No	Primary tumor	12	-	5	(Arredouani et al., 2009)
					Normal prostate (BPH)	7		1	
			GSE3325	No	Metastasis	6	Liver, lymph-node, lung, dura, soft tissue	2	(Varambally et al., 2005)
					Primary tumor	7		2	
					Normal prostate (BPH)	6		2	
			GSE45016	Yes	Metastatic primary tumor	7	Unknown	1	(Satake et al., 2010)
					Primary tumor	3		0	
					Normal prostate (BPH)	1		0	
		HG U133A 2.0	GSE6956	Yes	Primary tumor	69	-	69	(Wallace et al., 2008)
					Normal (NAD)	18		18	
					Normal prostate	2		2	
		HG U133A U133B	E-TABM-26	Yes	Primary tumor	44 × 2	-	72	(Liu, 2006)
					Normal (NAD)	13 × 2		17	
		HG U133A	GSE8218	No	Primary tumor	24	-	8	(Wang et al., 2010)
					Normal (NAD)	82		19	
			GSE32269	Yes	Metastasis	29	Bone	3	(Cai et al., 2013)
					Primary tumor	22		7	

		HG U95Av2 U95B U95C	GSE6919	Yes	Metastasis	24 × 3	Adrenal gland, kidney, lymph node, liver, lung	30	(Chandran et al., 2007)
					Primary tumor	63 × 3		138	
					Normal (NAD)	58 × 3		90	
					Normal prostate (BPH)	17 × 3		30	
		HG U95Av2	BI-GDAC	No	Primary tumor	52	-	34	(Singh et al., 2002)
					Normal (NAD)	50		27	
			GSE1431	No	Primary tumor	38	-	26	(Stuart et al., 2004)
					Normal (NAD)	50		5	

TCGA: The Cancer Genome Atlas; ICGC: International Cancer Genome Consortium; GA: Genome Analyzer; HuEx: Human Exon; HG: Human Genome; BI-GDAC: Broad Institute Genome Data Analysis Center; NAD: normal tissue adjacent to tumor; BPH: benign prostatic hyperplasia; HGPIN: high-grade prostatic intraepithelial neoplasia.

Table S2. Samples filtering steps and results.

Tissue type	Samples no.	Removed at (remained after) QC		
		Step 1*	Step 2	Step 3**
**Normal prostate & NAD**	795	168 (627)	0 (627)	155 (472)
HGPIN	49	3 (46)	0 (46)	13 (33)
Primary tumor	1,409	220 (1,189)	117 (1,072)	192 (880)
Metastatic primary tumor	36	4 (32)	0 (32)	1 (31)
Metastasis	107	31 (76)	4 (72)	0 (72)
	2,396	426 (1,970)	121 (1,849)	361 (1,488)

Step 1 - samples filtering based on initial quality control; Step 2 - samples filtering based on estimated tumor tissue content (< 40%); Step 3 - samples filtering based on principal component analysis and clustering; * Datasets GSE22260 and GSE32448 we entirely discarded due to overall poor quality; ** Datasets GSE6956 and E-TABM-26 were entirely discarded due to unexplained data variation; NAD: normal tissue adjacent to tumor; HGPIN: high-grade prostatic intraepithelial neoplasia.

Table S3. TaqMan gene expression assays used in this study (all Applied Biosystems).

Assay	Catalogue
ABCC5	Hs00981089_m1
AMACR	Hs01091292_m1
ATG5	Hs00355492_m1
CCNB2	Hs1084593_g1
CCNE1	Hs01026536_m1
CDC6	Hs00154374_m1
CDH1	Hs01023895_m1
CFL2	Hs01071313_g1
EFR3A	Hs00921359_m1
EVA1C	Hs00332708_m1
FHL1	Hs00793641_g1
INSM1	Hs00357871_s1
LGR4	Hs00173908_m1
MPZL1	Hs00535799_s1
MYC	Hs00153408_m1
NCOA2	Hs00896106_m1
NETO2	Hs00983152_m1
NEXN	Hs00936725_g1
NUP210	Hs00227779_m1
PID1	Hs00952182_m1

PLK1	Hs00983227_m1
RASAL2	Hs00183129_m1
SELENOM	Hs00369741_m1
SH2B2	Hs00184134_m1
SLC35A5	Hs00215733_m1
SYNPO2	Hs00326493_m1
TCERG1	Hs00198676_m1
TP63	Hs00978340_m1
ZCCHC6	Hs00226352_m1
PCDH18	Hs01556218_1
PGM5	Hs00222671_m1
FRMD6	Hs0078563_m1
PARM1	Hs00209876_m1
MKI67	Hs04260396_g1
MELK	Hs01106438_m1
NUSAP1	Hs01006195_m1
CENPF	Hs01118845_m1
TOP2A	Hs01032137_m1
GSTM2	Hs00265266_g1
GSTP1	Hs00943350_g1
CYP3A5	Hs02511768_s1
EZH2	Hs0054430_m1
ACSS3	Hs00998517_m1
MNX1	Hs00907365_m1
CYP27A1	Hs01017992_g1
YEATS2	Hs00216001_m1
DBT	Hs01066445_m1
PCCB	Hs00981334_m1
EYA4	Hs01012399_m1
GNAL	Hs00181836_m1
SH3BGRL2	Hs00230283_m1
C15ORF41	Hs01029996_m1
SLC25A3	Hs01390366_m1
MRFAP1	Hs00738144_g1
